# Polycyclic Aromatic Hydrocarbons (PAHs) in Interstellar Ices: A Computational Study into How the Ice Matrix Influences the Ionic State of PAH Photoproducts

**DOI:** 10.1021/acsearthspacechem.1c00433

**Published:** 2022-02-21

**Authors:** Stephanie ten Brinck, Celine Nieuwland, Angela van der Werf, Richard M. P. Veenboer, Harold Linnartz, F. Matthias Bickelhaupt, Célia Fonseca Guerra

**Affiliations:** † Department of Theoretical Chemistry, Amsterdam Institute of Molecular and Life Sciences (AIMMS), Amsterdam Center for Multiscale Modeling (ACMM), Vrije Universiteit Amsterdam, De Boelelaan 1083, 1081 HV Amsterdam, The Netherlands; ‡ Laboratory for Astrophysics, Leiden Observatory, Leiden University, P.O. Box 9513, 2300 RA Leiden, The Netherlands; § Institute for Molecules and Materials (IMM), Radboud University, Heyendaalseweg 135, 6525 AJ Nijmegen, The Netherlands; ∥ Leiden Institute of Chemistry, Gorlaeus Laboratories, Leiden University, Einsteinweg 55, 2333 CC Leiden, The Netherlands

**Keywords:** astrochemistry, density functional theory, charge-transfer excitations, ice-matrix effects, photoproducts, polycyclic aromatic hydrocarbons (PAHs)

## Abstract

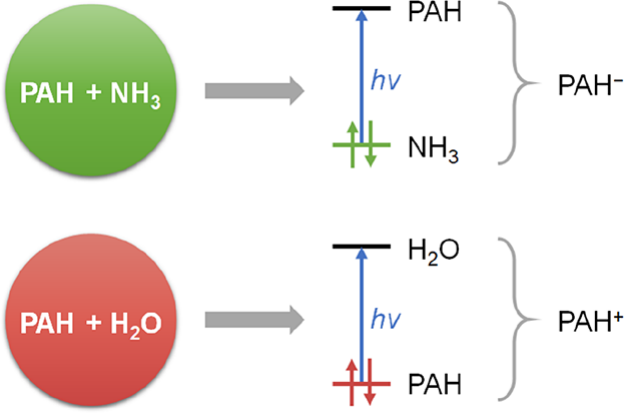

It has been experimentally observed that water–ice-embedded polycyclic aromatic hydrocarbons (PAHs) form radical cations when exposed to vacuum UV irradiation, whereas ammonia-embedded PAHs lead to the formation of radical anions. In this study, we explain this phenomenon by investigating the fundamental electronic differences between water and ammonia, the implications of these differences on the PAH–water and PAH–ammonia interaction, and the possible ionization pathways in these complexes using density functional theory (DFT) computations. In the framework of the Kohn–Sham molecular orbital (MO) theory, we show that the ionic state of the PAH photoproducts results from the degree of occupied–occupied MO mixing between the PAHs and the matrix molecules. When interacting with the PAH, the lone pair-type highest occupied molecular orbital (HOMO) of water has poor orbital overlap and is too low in energy to mix with the filled π-orbitals of the PAH. As the lone-pair HOMO of ammonia is significantly higher in energy and has better overlap with filled π-orbitals of the PAH, the subsequent Pauli repulsion leads to mixed MOs with both PAH and ammonia character. By time-dependent DFT calculations, we demonstrate that the formation of mixed PAH–ammonia MOs opens alternative charge-transfer excitation pathways as now electronic density from ammonia can be transferred to unoccupied PAH levels, yielding anionic PAHs. As this pathway is much less available for water-embedded PAHs, charge transfer mainly occurs from localized PAH MOs to mixed PAH–water virtual levels, leading to cationic PAHs.

## 1 Introduction

The presence of polycyclic aromatic hydrocarbons (PAHs) in the interstellar medium has been proven through the presence of strong infrared (IR) emission bands between 3.3 and 11.3 μm.^1^ For quite some time, these IR emission features could not be explained and therefore were named the unidentified IR features. In the 80s, these features were hypothesized to originate from various stretching and bending modes of vibrationally excited hydrocarbon systems, such as PAHs, after excitation by photons from the interstellar radiation field.^1b−1d,1g,2^ Nowadays, the existence of interstellar PAHs is generally accepted, specifically after the recent unambiguous astronomical identification of a number of aromatic species.^3^ Organic molecules such as PAHs play an important role in interstellar chemistry. They are formed in the outflows of dying stars, contribute upon fragmentation to the molecular inventory in space, and are expected to freeze out onto cold dust grains. Here, they get embedded in an ice matrix comprising mainly water but that also comprises other species that are expected to form upon the solid-state hydrogenation of atomic precursors, such as ammonia.^4,5^ Although water–ice is the main component of interstellar ice analogues, observations with the Spitzer Space Telescope showed that ammonia can be present in water-rich ices, with an abundance typically 10–20 times lower with respect to water.^6^ Upon exposure to high-energy irradiation, reactions of PAHs or PAH derivatives with the surrounding matrix are expected to occur. In fact, the vacuum UV (VUV) irradiation and particle bombardment of these interstellar ices produces complex organic molecules that possibly played a vital role in the origin of life.^7^

Previous *in situ* spectroscopic studies have shown that PAHs embedded in a water–ice matrix are readily ionized.^8,9^ For example, Gudipati and Allamandola showed that 4-methylpyrene, naphthalene, and quaterrylene in water–ice can be ionized by Lyman-α (10.2 eV) irradiation to their cationic forms.^8^ Similarly, Kofman *et al.* and Bouwman *et al.* observed the formation of cationic triphenylene and pyrene, respectively, in water–ice at 20 K when exposed to VUV broadband irradiation (120–160 nm) by recording the time-resolved electronic spectra of these PAHs as a function of fluence.^9,10^ In the study of Bouwman *et al.*, the authors state that the photolysis of pyrene is the result of direct single-photon ionization of the neutral species. In 2012, however, Cuylle *et al.* showed that both pyrene and benzo[*ghi*]perylene embedded in ammonia–ice become anionic when exposed to Lyman-α irradiation.^11^ The formation of anionic PAHs becomes more prevalent as the ratio of ammonia to water in mixed ices increases. Cuylle *et al.* hypothesized that an electron can be transferred to the PAH through ammonia-related photoproducts, and therefore, anionic PAHs are formed. They did, however, not provide experimental evidence for how this process occurs.

The purpose of our study is to achieve an understanding of the underlying physical factors responsible for the observation that upon VUV irradiation, the PAHs in water–ice turn into their radical cations, whereas the PAHs in ammonia–ice rather transform into the corresponding radical anions. Under interstellar and laboratory conditions, PAHs are fully surrounded by matrix molecules. Herein, we aim at disentangling the origin of the observed phenomena from other bulk effects and investigate if, already at the level of the interaction between a single representative PAH and a single matrix molecule,^12^ the two opposite tendencies can be recovered and related to a causal physical mechanism.

To understand the effect of the matrix on the photochemical behavior of the PAH in its essence, we need to go back to the fundamental difference in the interaction between one PAH molecule and one water *versus* one ammonia molecule. We first quantum-chemically analyze the fundamental differences between the electronic structures of water and ammonia in relation to those of three representative PAHs, benzene (**Ben**, C_6_H_6_), pyrene (**Py**, C_16_H_10_), and benzo[*ghi*]perylene (**BgP**, C_22_H_12_), using Kohn–Sham molecular orbital (MO) theory, as contained in Kohn–Sham density functional theory (DFT). Second, we analyze how these differences affect the nature of the PAH–water *versus* PAH–ammonia interaction with respect to the MO electronic spectra of the resulting PAH–matrix complexes and the associated potential ionization routes. Third, we compute the actual excitations and their oscillator strength using time-dependent DFT (TDDFT).

Here, we anticipate that we find fundamental differences between water and ammonia molecules that translate into opposite charge-transfer excitations in the corresponding complexes with PAHs, leading preferentially to PAH-to-water excitation (*i.e.*, PAH cation formation in the case of water) and ammonia-to-PAH excitation (*i.e.*, PAH anion formation in the case of ammonia).

## 2 Computational Details

### 2.1 General Procedure

Calculations were carried out with the Amsterdam Density Functional (ADF) program, version 2013.01.^13^ The generalized gradient approximation functional BLYP was employed to find the equilibrium structures of all species using the basis set TZ2P, a small frozen core, and Grimme’s DFT-D3 correction with Becke–Johnson (BJ) damping to correct for dispersion effects.^14^ The TZ2P basis set is of valence triple-ζ quality and has been augmented by two polarization functions.^15^ This basis set is known from previous work to yield relatively small basis-set superposition errors that can be safely neglected; see ref (15b). Scalar relativistic corrections were included using the zeroth-order regular approximation (ZORA) to allow for the possible expansion of this work in the future.^16,17^ Numerical integration was performed with the Becke grid set to “verygood”.^18^ All geometry optimizations were performed in the gas phase and without imposing geometric constraints. The equilibrium geometries were confirmed to be in a (local) minimum-energy state using vibrational frequency analyses (*i.e.*, no imaginary frequencies were found).

### 2.2 Fock Matrix-Symmetrized Fragment Orbitals (FMATSFOs)

In the ADF program, the electronic structure of molecular systems, such as our PAH–matrix complexes, can be analyzed in terms of the interaction between two meaningfully chosen fragments and the bonding mechanism between the corresponding fragment MOs. The effective orbital energies of fragments can and, in general, do change once these fragments are at their positions in the final complex and experience each other’s electrostatic potential.^19,20^ Often, but not always, the effective fragment MO energies are stabilized when they are exposed to the potential of the other fragment. Note that this is the actual starting situation from which orbital interactions take place. These shifted effective fragment orbital energies can be computed in ADF using the keyword FMATSFO.^19^

### 2.3 TDDFT Computations

Possible charge-transfer excitations between the PAHs and matrix molecules were investigated using TDDFT.^21^ All optically allowed (*i.e.*, singlet–singlet) vertical excitations were calculated for the PAH–water and the PAH–ammonia complexes. The range-separated functional CAMY-B3LYP was used, along with a TZ2P basis set.^22^ Relativistic effects were accounted for using ZORA.^16,17^ The CAMY-B3LYP functional has shown to provide accurate results for charge-transfer excitations in naphthalene derivatives and is therefore a suitable functional to study the PAHs in this work.^23^ For more detailed information on the CAMY-B3LYP functional, we refer to ref (22b).

## 3 Results and Discussion

### 3.1 MO Analysis of Ammonia, Water, and PAHs

**Figure 1:**
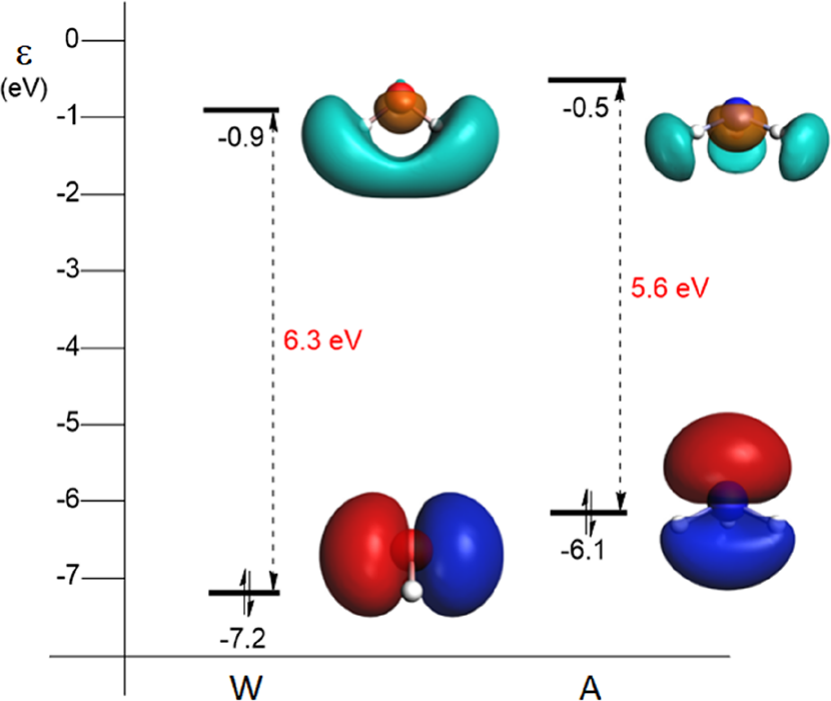
HOMO and LUMO energy levels (in eV) of water (**W**) and ammonia (**A**), along with their isosurfaces (at 0.05 au), calculated at the ZORA-BLYP-D3(BJ)/TZ2P level of theory in the gas phase.

Understanding of the ionization pathways of PAHs in water and ammonia–ice starts with the quantum chemical analysis of the electronic structure of the two different-matrix molecules (H_2_O and NH_3_). In this analysis, we focus on the highest occupied MOs (HOMOs) and lowest unoccupied MOs (LUMOs) in particular since these levels are most likely involved in electronic excitations. Figure 1 shows the optimized geometries and the HOMO and LUMO orbitals and corresponding energies of water and ammonia.

The HOMO of ammonia (HOMO_**A**_) is at a relatively high energy (−6.1 eV) compared to the HOMO of water (HOMO_**W**_, −7.2 eV), resulting in an energy difference between the two HOMOs of 1.1 eV. These HOMOs represent the lone pair (LP) of ammonia and the highest-energy LP of water (the HOMO – 1_**W**_ of water, i.e., the other LP, lies at −9.2 eV). In contrast to the HOMOs, the energy difference between the LUMOs of the two matrix molecules is relatively small, only 0.4 eV, where the LUMO of ammonia lies at −0.5 eV and the LUMO of water at −0.9 eV. Qualitatively, similar results are obtained when the water and ammonia molecule are in a medium of other like molecules (the HOMO–LUMO gaps increase by *ca.* 0.5 eV; see Figure S1).

**Figure 2:**
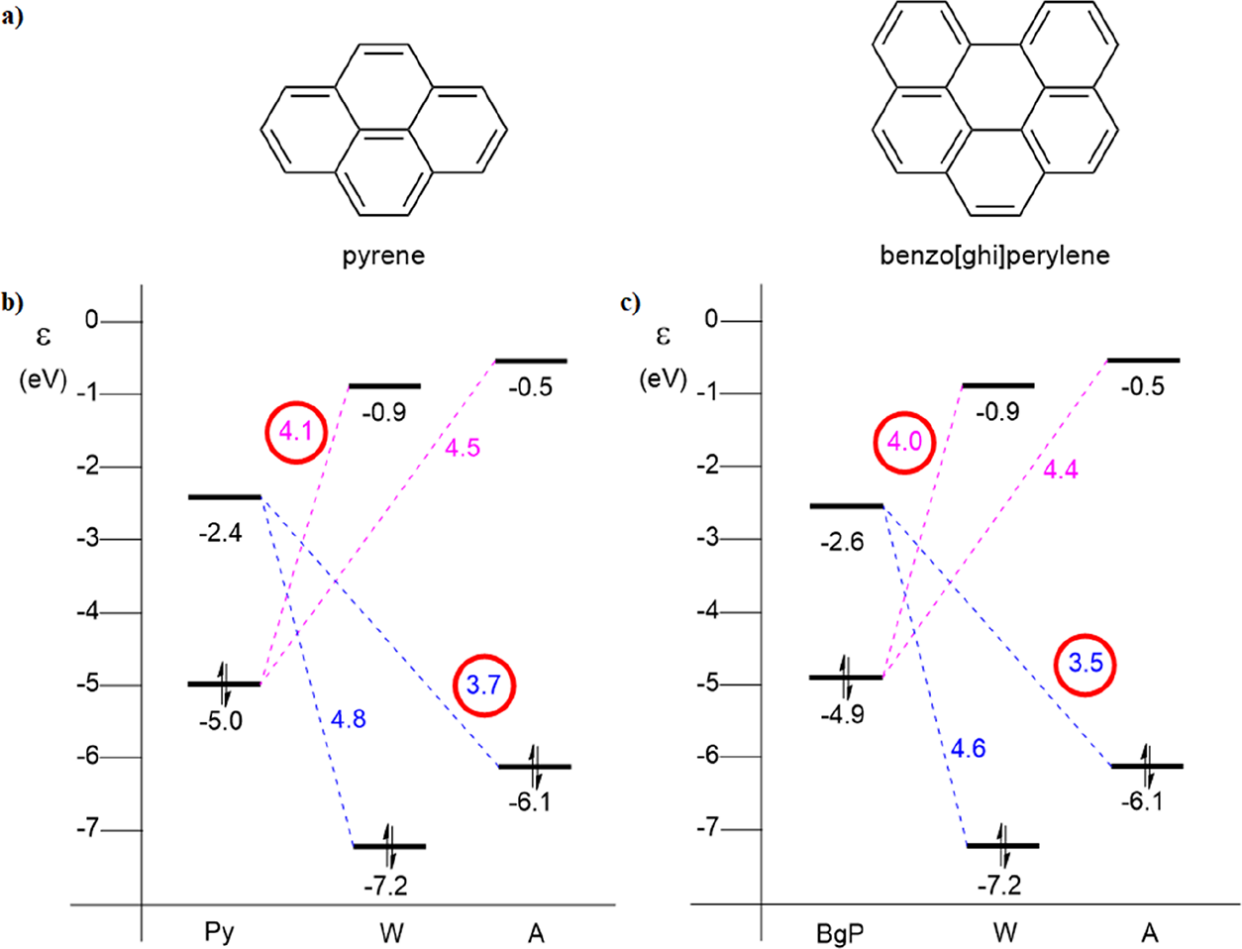
(a) Molecular structures of pyrene (**Py**) and benzo[*ghi*]perylene (**BgP**). (b) HOMO–LUMO energy levels (in eV) of water (**W**) and ammonia (**A**) compared to **Py** and (c) **BgP**, calculated at the ZORA-BLYP-D3(BJ)/TZ2P level of theory in the gas phase. The smallest intermolecular energy gaps are encircled in red.

Next, we compare the relative positioning of the HOMO and LUMO of ammonia and water to the frontier MOs of pyrene (**Py**) and benzo[*ghi*]perylene (**BgP**), which are representative for the PAHs used in the experiments by Cuylle *et al.* [see Figure 2 for the energy levels of **Py**, **W**, and **A** (left) and **BgP**, **W**, and **A** (right)].^11^ The HOMO and LUMO have π- and π*-like characters, respectively, for both **Py** and **BgP**. The smallest intermolecular HOMO–LUMO gap with regard to water and ammonia is encircled in red (see Figure 2). The energy gap between the HOMO_**A**_ and the LUMO_**PAH**_ is smaller than the energy gap between the HOMO_**W**_ and the LUMO_**PAH**_ due to the higher-lying HOMO_**A**_. For ammonia, it amounts to 3.7 and 3.5 eV between HOMO_**A**_ and, respectively, the LUMO_**Py**_ and LUMO_**BgP**_. For water, the values are 4.8 and 4.6 eV between the HOMO_**W**_ and, respectively, the LUMOs of **Py** and **BgP**. The energy differences between the HOMO of the PAHs and the LUMOs of ammonia and water are less pronounced as the LUMOs of the matrix molecules differ less. The smallest energy gap here is found between the HOMO_**PAH**_ and the LUMO_**W**_, being 4.1 and 4.0 eV for **Py** and **BgP**, respectively (see also Figure 2).

Let us consider an electron transfer in these model systems assuming that the electron transfer occurs *via* the smallest energy gap (see encircled gaps in Figure 2). In a PAH–water complex, the electron is excited over the smaller energy gap from the PAH to water (4.1 and 4.0 eV for **Py** and **BgP**) than from water to the PAH (4.8 and 4.6 eV for **Py** and **BgP**). This suggests that it is more likely to form cationic PAHs in a PAH–water complex than the anionic species. In the case of PAH–ammonia complexes, it is the other way around. The electron is more easily excited over the smaller energy gap from ammonia to the PAH (3.7 and 3.5 eV for **Py** and **BgP**, respectively) than from the PAH to ammonia (4.5 and 4.4 eV for **Py** and **BgP**, respectively). Here, the suggestion is that it is more likely to form anionic PAHs in a PAH–ammonia complex than cationic PAHs. These findings are consistent with the observations of Cuylle *et al.* in their *in situ* experiments.^11^

The ground-state energetics of the individual molecules give a good indication of the electronic differences between ammonia and water and how the relative HOMO and LUMO energies of ammonia, water, and the PAHs are responsible for the size of the intermolecular energy gaps. Note, however, that this simplified picture is an incomplete representation as charge-transfer excitations that occur in interstellar ices involve PAHs embedded in them, thus interacting with ammonia and water. The individual ground-state HOMO–LUMO levels do not take into account any change in the MOs as a result of the interaction between the PAHs and the matrix molecules. This coupling or mixing of the PAH levels with the matrix electronic structure is crucial for understanding the actual factors determining the transition probability for PAHs in water–ice *versus* that of PAHs in ammonia–ice (*vide infra*).

### 3.2 MO Interaction Diagrams of PAH–Matrix Complexes

**Figure 3:**
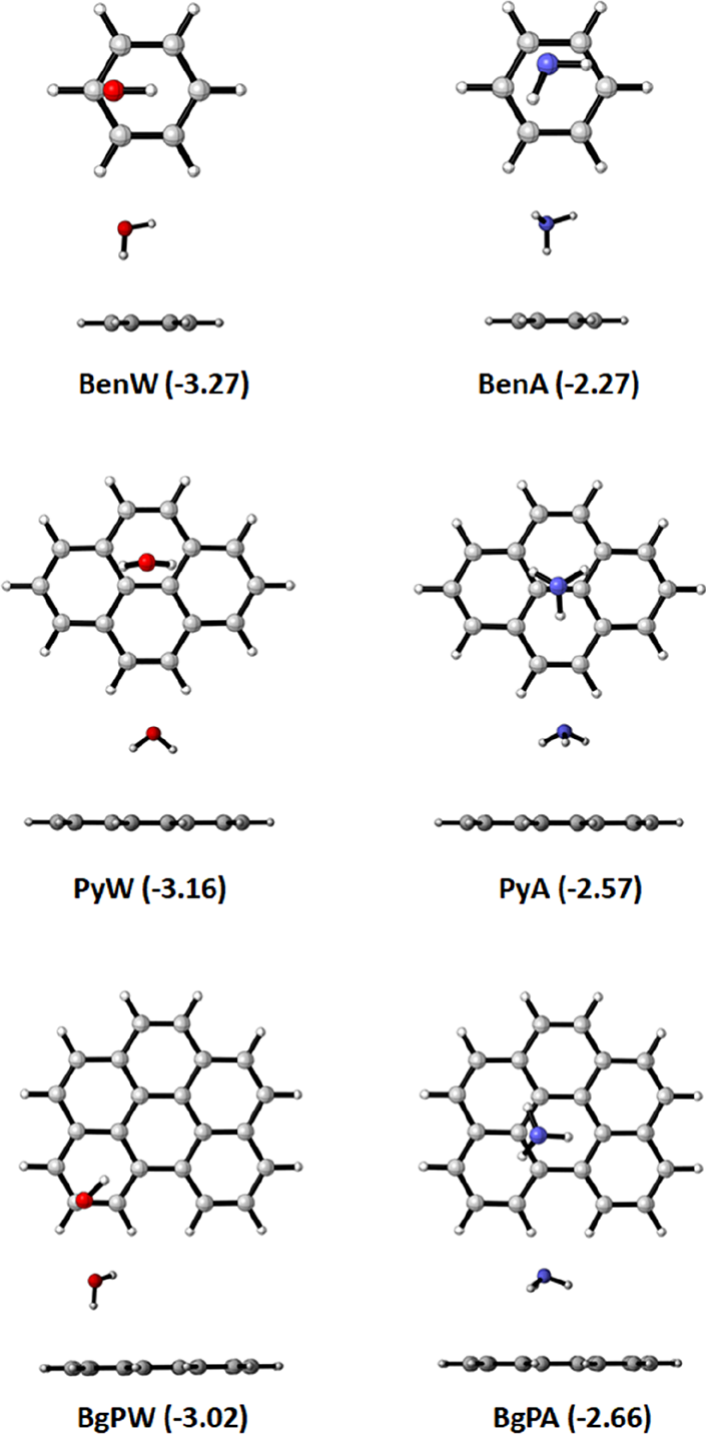
Optimized PAH–matrix complexes (top view and side view) with the calculated bond energy shown in parentheses in kcal mol^–1^, calculated at the ZORA-CAMY-B3LYP-D3(BJ)/TZ2P//ZORA-BLYP-D3(BJ)/TZ2P level of theory. For the bond energy calculated fully with ZORA-BLYP-D3(BJ)/TZ2P, see Figure S2.

In the previous section, we analyzed the ground-state frontier MOs of the individual PAH, water, and ammonia molecules. For a basic analysis of the MOs of small molecules, we refer the reader to the book “Orbital Interactions in Chemistry” by T. A. Albright *et al.*, see ref (24). Next, we quantify the interactions in the PAH–ammonia and PAH–water complexes to see how the HOMO–LUMO levels change as a result of the interaction. Interacting PAH–matrix complexes were created by placing a single matrix molecule above the π-electronic system of the aromatic rings and subsequently optimizing the complex (see Figure 3). For each of the final six complexes (labeled **BenW**, **BenA**, **PyW**, **PyA**, **BgPW**, and **BgPA**), energy minima were found. In the optimized geometries, the ammonia and water molecules direct the N–H or O–H bond(s) toward the π-system of the PAH, see Figure 3. The Cartesian coordinates of the optimized PAH–matrix complexes can be found in the Supporting Information. Note that we find several local minima for each PAH–matrix combination. These complexes are very similar, both energetically and in terms of their interaction mechanism but only differ in the exact position at which the matrix molecule binds to the π-system of the PAH. Herein, we highlight one of the obtained complexes per PAH–matrix combination that clearly demonstrates the fundamental differences between the PAH–ammonia and PAH–water complexes. For the geometries of the other computed PAH–matrix complex minima, the reader is referred to Figure S3.

The interaction between the PAH and matrix molecule when forming the PAH–matrix complexes is small, with the bond energies ranging between −2.27 and −3.27 kcal mol^–1^, which can be considered as a weak N(H)···π or O(H)···π hydrogen bond interaction. In general, the bond energy for the PAH–water complexes (−3.02 to −3.27 kcal mol^–1^) is more stabilizing than that for the PAH–ammonia complexes (−2.27 to −2.66 kcal mol^–1^).

Next, the interaction between the PAH and the matrix molecules in the PAH–matrix complexes is further investigated. The **Py**–matrix complexes are highlighted here to provide general insights on the PAH–matrix interaction as similar results were found for the **Ben**–matrix and **BgP**–matrix complexes. Additional orbital interaction figures for the **Ben**–matrix, **Py**–matrix, and **BgP**–matrix complexes can be found in Figures S4–S9 of the Supporting Information.

**Figure 4:**
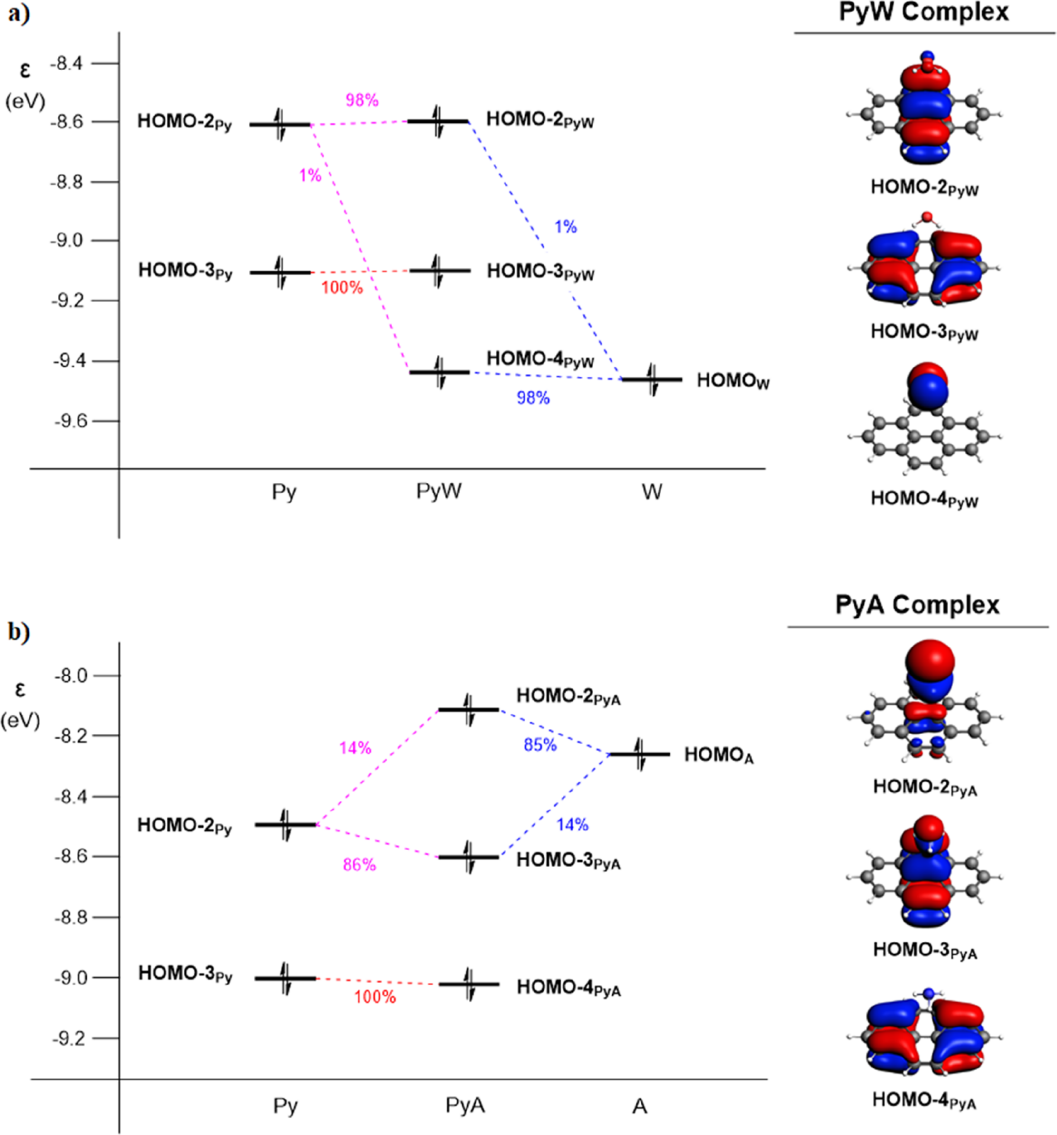
Orbital interaction diagram of occupied fragment MOs (FMOs) of (a) **PyA** and (b) **PyW**, with FMO contributions (in %) and visualization of the overall complex MOs (isosurface at 0.03 au), calculated at the ZORA-CAMY-B3LYP/TZ2P level of theory. The FMO energies are calculated in the field of the other fragment, as described in Computational DetailsSection 2.2, and reported in Table S3. For a full description of the occupied MOs, see Figures S6 and S7.

The interaction between the occupied MOs of pyrene and the matrix molecules water and ammonia is given in Figure 4. The extent of mixing between the PAH and matrix-molecule orbitals is determined not only by the differences in energy between the orbitals of the PAHs and the matrix molecules but also for an important extent by the orbital overlap. Now, we first inspect how much the extent of mixing is in the case of a water and an ammonia matrix. For the **PyW** complex (Figure 4a), there is very little-to-no mixing that occurs in the occupied MOs of the **PyW** complex, with roughly 98% of the HOMO – 2_**PyW**_ originating from the HOMO – 2_**Py**_, 100% of the HOMO – 3_**PyW**_ originating from the HOMO – 3_**Py**_, and 98% of the HOMO – 4_**PyW**_ originating from the HOMO_**W**_. Thus, the occupied π-orbitals of pyrene (HOMO – 2_**Py**_ and HOMO – 3_**Py**_) and the LP of water (HOMO_**W**_) mix minimally and remain mostly localized on the original fragments.

In contrast, the LP of ammonia (HOMO_**A**_) and a π-orbital of pyrene (HOMO – 2_**Py**_) mix strongly in the **PyA** complex (Figure 4b). Two delocalized MOs (HOMO – 2_**PyA**_ and HOMO – 3_**PyA**_) are formed, with roughly 14% of the total MO originating from one monomer while the remaining 85–86% originating from the other monomer (see Figure 4b). It is a manifestation of the Pauli exclusion principle due to the overlap between occupied orbitals of both monomers (see Tables S1 and S2). The HOMO – 3_**PyA**_ is a bonding combination, and the HOMO – 2_**PyA**_ is an antibonding combination between the HOMO – 2_**Py**_ and the HOMO_**A**_. This difference in interaction between the PAH–water and PAH–ammonia complexes can be attributed to the HOMO energies of the two matrix molecules and orbital overlap between HOMO of the matrix molecules (ammonia or water) and π-orbitals of the PAH.

Now, we return to the rationale behind the stronger PAH–matrix orbital mixing in the case of ammonia compared to water. The amount of mixing between an orbital on one monomer and an orbital on the other monomer is approximately proportional to the overlap between the two orbitals on both monomers *S* (more precisely, it is proportional to the matrix element between the two orbitals) and inversely proportional to the energy difference between those orbitals, Δε. The absolute energy gap between the HOMO_**A**_ of ammonia and the HOMO – 2_**Py**_ (0.24 eV) is much smaller than the gap between the HOMO_**W**_ of water and the HOMO – 2_**Py**_ (0.84 eV), as can be seen in Figure 4. Furthermore, the spatial orientation of the LP of ammonia relative to the PAH allows for better orbital overlap with the PAH MOs in comparison to water (see also Figures 1 and 3). The reason for this is that the ammonia LP is essentially the nitrogen 2p_*z*_ (with a slight admixture of hydrogen 1s character), and due to ammonia’s trigonal pyramidal geometry, as it points with its N–H bond(s) to the PAH, the LP also points directly to the π-system of the PAH and, thus, overlaps well. In contrast, the oxygen 2p-type LP is arranged parallel to the PAH surface as the water molecule points with its O–H bond(s) to the PAH (see also Figures 1 and 3). This orbital does not point with a lobe toward the PAH and therefore has a smaller overlap with the π-system. For example, in the **PyA** complex, the orbital overlap between the LP of ammonia (HOMO_**A**_) and the π-orbital of pyrene (HOMO – 2_**Py**_) is larger (*S* = 0.0266) than the overlap between the LP of water (HOMO_**W**_) and the π-orbital of pyrene (HOMO – 2_**Py**_) in the **PyW** complex, which is only 0.0175 (see Table S2). The orbital overlap between the HOMO_**W**_ and HOMO – 3_**Py**_ is even 1 order of magnitude smaller (*S* = 0.0017). These smaller orbital overlaps explain why the MOs of pyrene do not mix significantly with the HOMO_**W**_.

The strong mixing in the **PyA** complex and the poor mixing in the **PyW** complex is a clear fundamental difference between these two complexes. As will be shown in the following section, the formation of these mixed PAH–ammonia MOs plays an important role in the available charge-transfer pathways.

### 3.3 Charge-Transfer Excitations in Interacting PAH–Matrix Complexes

**Figure 5:**
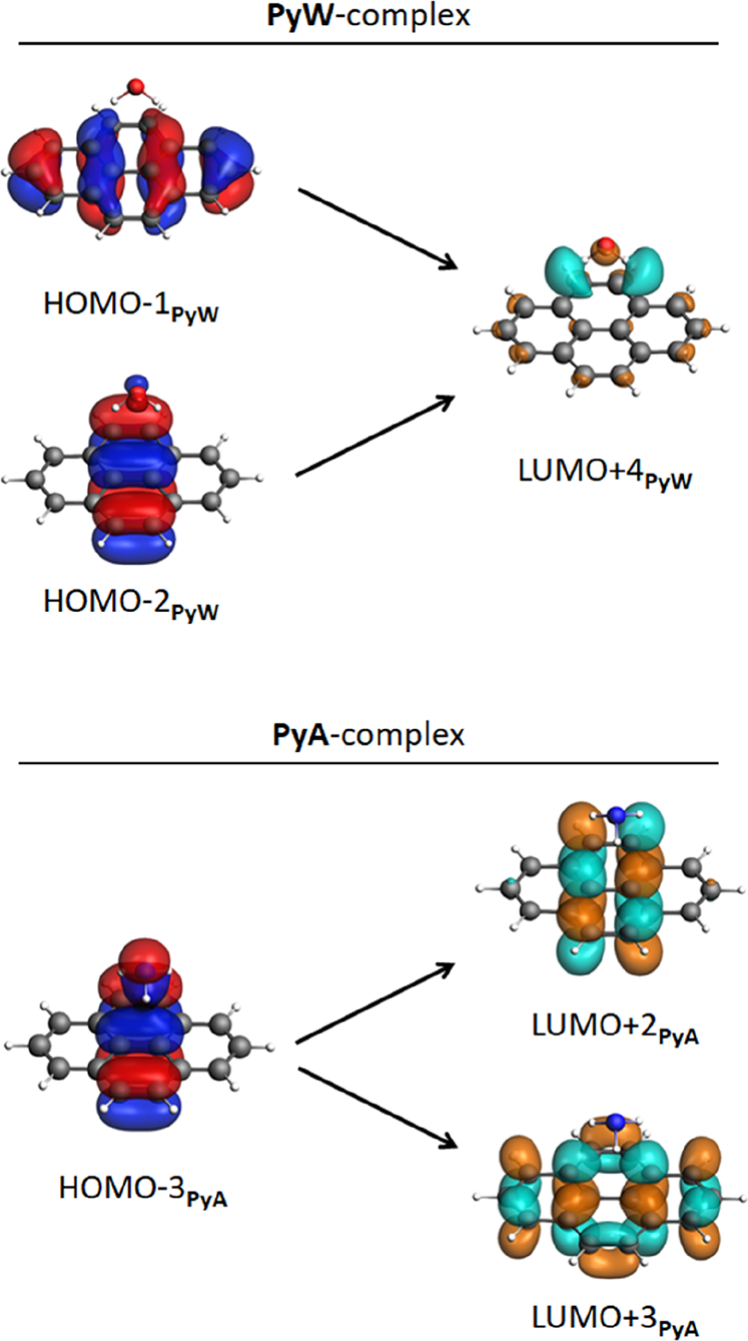
Relevant MOs involved in charge-transfer excitations for **PyW** (top) and **PyA** (bottom) with isosurfaces (at 0.03 au) calculated at the CAMY-B3LYP/TZ2P level of theory. Occupied MOs are red and blue, while unoccupied MOs are cyan and orange. Optimized structures can be found in Figure 3. The situation is similar for **BenW**, **BenA**, **BgPW**, and **BgPA**; see Figures S12 and S13.

In this section, we analyze the possible charge-transfer excitation pathways in the PAH–matrix complexes (Figure 3) using TDDFT calculations. All charge-transfer excitations below 10.2 eV (Lyman-α radiation) with an oscillator strength greater than 0.2 are considered in the exploration of the possibility of charge transfer upon VUV irradiation. The calculated charge-transfer excitations are reported in Table 1 for the complexes of Figure 3. See Table S4 and Figure S11 for the charge-transfer excitations of other local minima of these PAH–matrix complexes. In this section, we focus mostly on the **Py**–matrix complexes as these complexes clearly explain the difference between PAH–water and PAH–ammonia complexes. All relevant MOs involved in the **Py**–matrix excitations are shown in Figure 5. The MOs of the **Ben**–matrix and **BgP**–matrix complexes can be found in Figure S10.

**Table 1:** Charge-Transfer Excitations (in eV) in PAH−Matrix Complexes Calculated at the ZORA-CAMY-B3LYP/TZ2P Level of Theory^a^

complexes	transition (eV)	oscillator strength	weight (%)	from the MO (complex)	to the MO (complex)	character of charge-transfer direction
**BenW**	7.09	0.443	18.9	HOMO – 1_**BenW**_	LUMO + 2_**BenW**_	π_PAH_ → σ_PAH_^*^ +
**BenA**	7.11	0.610	26.4	HOMO – 2_**BenA**_	LUMO + 1_**BenA**_	π_PAH_ & LP_NH_3__ → π_PAH_^*^
			20.3	HOMO_**BenA**_	LUMO + 1_**BenA**_	π_PAH_ & LP_NH_3__ → π_PAH_^*^
	7.10	0.547	25.7	HOMO – 2_**BenA**_	LUMO_**BenA**_	π_PAH_ & LP_NH_3__ → π_PAH_^*^
			19.3	HOMO_**BenA**_	LUMO_**BenA**_	π_PAH_ & LP_NH_3__ → π_PAH_^*^
**PyW**	7.35	0.599	12.2	HOMO – 2_**PyW**_	LUMO + 4_**PyW**_	π_PAH_ → σ_PAH_^*^ +
	6.70	0.544	29.3	HOMO – 1_**PyW**_	LUMO + 4_**PyW**_	π_PAH_ → σ_PAH_^*^ +
	7.21	0.418	5.3	HOMO – 2_**PyW**_	LUMO + 4_**PyW**_	π_PAH_ → σ_PAH_^*^ +
	6.66	0.259	57	HOMO – 1_**PyW**_	LUMO + 4_**PyW**_	π_PAH_ → σ_PAH_^*^ +
**PyA**	7.30	0.780	62.3	HOMO – 3_**PyA**_	LUMO + 3_**PyA**_	π_PAH_ & LP_NH_3__ → π_PAH_^*^
	6.76	0.675	85.9	HOMO – 3_**PyA**_	LUMO + 2_**PyA**_	π_PAH_ & LP_NH_3__ → π_PAH_^*^
**BgPW**	6.53	1.053	5.7	HOMO – 1_**BgPW**_	LUMO + 5_**BgPW**_	π_PAH_ → σ_PAH_^*^ +
			3.7	HOMO_**BgPW**_	LUMO + 8_**BgPW**_	π_PAH_ → σ_PAH_^*^ +
**BgPA**	6.95	0.742	31.0	HOMO – 3_**BgPA**_	LUMO + 4_**BgPA**_	π_PAH_ & LP_NH_3__ → π_PAH_^*^
	6.58	0.443	26.7	HOMO – 3_**BgPA**_	LUMO + 3_**BgPA**_	π_PAH_ & LP_NH_3__ → π_PAH_^*^
			8.4	HOMO – 2_**BgPA**_	LUMO + 3_**BgPA**_	π_PAH_ & LP_NH_3__ → π_PAH_^*^
	6.46	0.353	38.6	HOMO – 2_**BgPA**_	LUMO + 3_**BgPA**_	π_PAH_ & LP_NH_3__ → π_PAH_^*^
	6.60	0.268	15.1	HOMO – 3_**BgPA**_	LUMO + 3_**BgPA**_	π_PAH_ & LP_NH_3__ → π_PAH_^*^
	6.13	0.205	23.3	HOMO – 2_**BgPA**_	LUMO + 2_**BgPA**_	π_PAH_ & LP_NH_3__ → π_PAH_^*^

a Lone pairs are abbreviated as LP. The MOs involved in the charge-transfer excitations (columns 5 and 6) can be found in Figure 5.

In the **PyW** complex, charge-transfer excitations can occur from the localized π-orbitals of pyrene (HOMO – 1_**PyW**_ or HOMO – 2_**PyW**_) to a delocalized unoccupied MO consisting of the mixed PAH–water σ*-orbital with a large coefficient on the water molecule (LUMO + 4_**PyW**_), see Figure 5. This direction of charge-transfer excitations suggests that pyrene donates electronic density to water and therefore can become positively charged upon irradiation.

Interestingly, the direction of charge-transfer excitations in the **PyA** complex is different from the water-embedded variant **PyW**. In the **PyA** complex, charge-transfer excitations are observed from a mixed MO with ammonia and pyrene π-orbital character (HOMO – 3_**PyA**_) to two different unoccupied MOs with a π*-like character localized on the pyrene molecule (LUMO + 2_**PyA**_ or LUMO + 3_**PyA**_), see Figure 5. This direction of charge-transfer excitation leads to a shift of charge density from the occupied mixed MO of the complex toward the unoccupied orbitals of pyrene and indicates a net electronic density flow from ammonia to pyrene, leading to a negatively charged PAH upon irradiation. These results on the dimers of PAH–water and PAH–ammonia show the same trends as the experimental findings by Cuylle *et al.*, who reported the formation of anionic PAHs in an ammonia matrix, while cationic PAHs were formed in a water matrix.^11^

The results for the **BenW** and **BgPW** complexes are similar to those for the **PyW** complex: charge-transfer excitations are observed from localized π-orbitals (HOMO – 1_**BenW**_ for **BenW** and HOMO_**BgPW**_ or HOMO – 1_**BgPW**_ for **BgPW**) toward mixed PAH–water σ*-MOs (LUMO + 2_**BenW**_ for **BenW** and LUMO + 5_**BgPW**_ or LUMO + 8_**BgPW**_ for **BgPW**). This suggests the formation of cationic PAH radicals upon the irradiation of PAH–water complexes. For the **BenA** and **BgPA** complexes, all observed charge-transfer excitations originate from mixed MOs of the LP of ammonia and the π-orbitals of the aromatic molecule (HOMO_**BenA**_ or HOMO – 2_**BenA**_ for **BenA** and HOMO – 2_**BgPA**_ or HOMO – 3_**BgPA**_ for **BgPA**) toward localized π*-orbitals of the aromatic molecule (LUMO_**BenA**_ or LUMO + 1_**BenA**_ for **BenA** and LUMO + 2_**BgPA**_, LUMO + 3_**BgPA**_, or LUMO + 4_**BgPA**_ for **BgPA**). Accordingly, this direction of charge transfer suggests the formation of anionic PAH radicals. These results are recovered in all other PAH–ammonia complexes, which all go with predominant ammonia to PAH excitations. In the case of other PAH–water complexes, the situation is not always that consistent, and in some cases, besides PAH-to-water excitations, there also exist water-to-PAH excitations.

In the study by Cuylle *et al.*, it was shown that changing the ratio of water/ammonia in the ice matrix gives rise to a turning point in the ionization direction, that is, raising the ratio water/ammonia led to the formation of cationic PAHs over anionic PAHs. Our TDDFT findings nicely explain this phenomenon: raising the ratio water/ammonia implies strengthening the mechanism that leads to PAH cation formation while lowering the ratio water/ammonia corresponds to reinforcing the mechanism of PAH anion formation. Note however that our archetypal model systems do not allow for a prediction of the water/ammonia ratio at which the turning point occurs.

## 4 Conclusions

We have quantum-chemically developed a rationale for the experimental observation that water–ice-embedded PAHs become cationic when exposed to high-energy irradiation, whereas ammonia–ice-embedded PAHs lead to the formation of anionic PAH photoproducts. Interestingly, this turns out to be not so much an average medium effect but rather the consequence of discrete quantum chemical interactions that are already effective upon bonding one matrix molecule in PAH–water and PAH–ammonia complexes.

Our analyses, based on Kohn–Sham MO theory and TDDFT, reveal that the nitrogen LP-type HOMO of ammonia is optimally oriented toward the aromatic π-system as this matrix molecule binds with its N–H bond(s) to the PAH. This orientation, and the relatively high energy of the ammonia LP HOMO, guarantees good overlap and strong mixing between occupied ammonia and PAH orbitals. Indeed, our TDDFT analyses confirm that excitations from occupied orbitals of the PAH–ammonia complex have a strong component of charge transfer from the ammonia matrix into virtual orbitals that are largely on the PAH. In other words, this generates PAH radical anions.

At variance, the oxygen LP-type HOMO of water is suboptimally oriented, namely, in parallel to the aromatic π-system as this matrix molecule binds with its O–H bond(s) to the PAH. This orientation, and the somewhat lower energy of the water LP HOMO, impair the buildup of overlap and the mixing between occupied water and PAH orbitals. This yields a situation in which excitations take place from occupied orbitals that remain mainly localized on the PAH into virtuals with water and PAH character, as perceived from TDDFT analyses. This comes down to generating PAH radical cations.

## Supporting Information Available

Bond energies, orbital interaction diagrams, and MOs of the PAH–matrix complexes and energy decomposition analysis, TDDFT results, and Cartesian coordinates of the optimized PAH–matrix complexes (PDF)
