# Supplementary material for: Polycyclic Aromatic Hydrocarbons (PAHs) in Interstellar Ices: A Computational Study into How the Ice Matrix Influences the Ionic State of PAH Photoproducts
Source: ACS Earth Space Chem. 2022 Feb 21;6(3):766–74. doi: 10.1021/acsearthspacechem.1c00433 (PMC8935472; doi:10.1021/acsearthspacechem.1c00433)
Supplement: Supplementary file 1 — sp1c00433_si_001.pdf [file sp1c00433_si_001.pdf]

Supporting Information for

PAHs in interstellar ices: A computational study into how the ice matrix influences the ionic state of PAH photoproducts

Stephanie ten Brinck[a], Celine Nieuwland[a], Angela van der Werf[a], Richard M. P. Veenboer[a], Harold Linnartz[b], F. Matthias Bickelhaupt[a,c*], and Célia Fonseca Guerra[a,d*]

[a]Department of Theoretical Chemistry, Amsterdam Institute of Molecular and Life Sciences (AIMMS), Amsterdam Center for Multiscale Modeling (ACMM), Vrije Universiteit Amsterdam, De Boelelaan 1083, 1081 HV Amsterdam, The Netherlands
[b]Laboratory for Astrophysics, Leiden Observatory, Leiden University, P.O. Box 9513, 2300 RA Leiden, The Netherlands
[c]Institute for Molecules and Materials (IMM), Radboud University, Heyendaalseweg 135, 6525 AJ Nijmegen, The Netherlands
[d]Leiden Institute of Chemistry, Gorlaeus Laboratories, Leiden University, Einsteinweg 55, 2333 CC Leiden, The Netherlands

Table of contents

Figure S1 | HOMO–LUMO energy of ammonia and water using COSMO

Figure S2 | Bond energies of the PAH–matrix complexes

Figure S3 | Bond energies of the extra PAH–matrix complexes

Figure S4 | Orbital interaction diagram of **BenW**

Figure S5 | Orbital interaction diagram of **BenA**

Figure S6 | Orbital interaction diagram of **PyW**

Figure S7 | Orbital interaction diagram of **PyA**

Figure S8 | Orbital interaction diagram of **BgPW**

Figure S9 | Orbital interaction diagram of **BgPA**

Figure S10 | Overview of molecular orbitals of the PAH–matrix complexes involved in charge transfer excitations

Figure S11 | Overview of molecular orbitals of the extra PAH–matrix complexes involved in charge transfer excitations

Figure S12 | Charge transfer excitations in **BenW** and **BenA**

Figure S13 | Charge transfer excitations in **BgPW** and **BgPA**

Supporting Method | Energy decomposition analysis (EDA)

Table S1 | Energy decomposition analysis of the PAH–matrix complexes

Table S2 | Orbital overlap in the PAH–matrix complexes

Table S3 | Fock Matrix Symmetrized Fragment Orbital (FMATSFO)

Table S4 | TDDFT results of the extra PAH–matrix complexes

Supporting Data | Cartesian coordinates of the optimized PAH–matrix complexes

Figure S1 | HOMO–LUMO energy of ammonia and water using COSMO

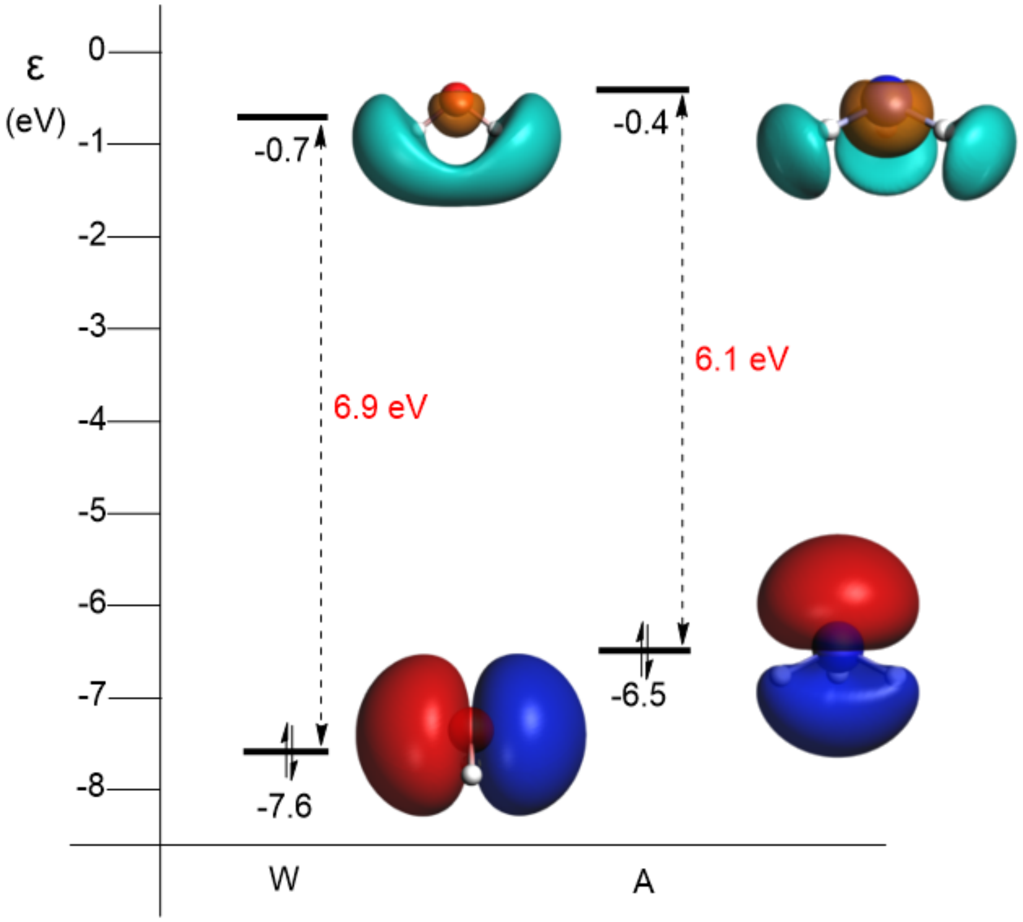

Figure S1 | HOMO and LUMO energy levels (in eV) of water (**W**) and ammonia (**A**), along with their isosurfaces (at 0.05 au), calculated at the ZORA-BLYP-D3(BJ)/TZ2P level of theory using the Conductor-like Screening Model (COSMO) with ADF2019.[1] COSMO allows for the inclusion of solvent effects for each molecule, with **W** in water and **A** in ammonia, respectively.

[1] **a)** Klamt, A.; Schüürmann, G. COSMO: A New Approach to Dielectric Screening in Solvents with Explicit Expressions for the Screening Energy and its Gradient. *J. Chem. Soc. Perkin Trans. 2,* **1993**, *5*, 799–805. **b)** Pye, C. C.; Ziegler, T.; An implementation of the conductor-like screening model of solvation within the Amsterdam density functional package. *Theor. Chem. Acc.* **1999**, *101*, 396–408.

Figure S2 | Bond energies of the PAH–matrix complexes

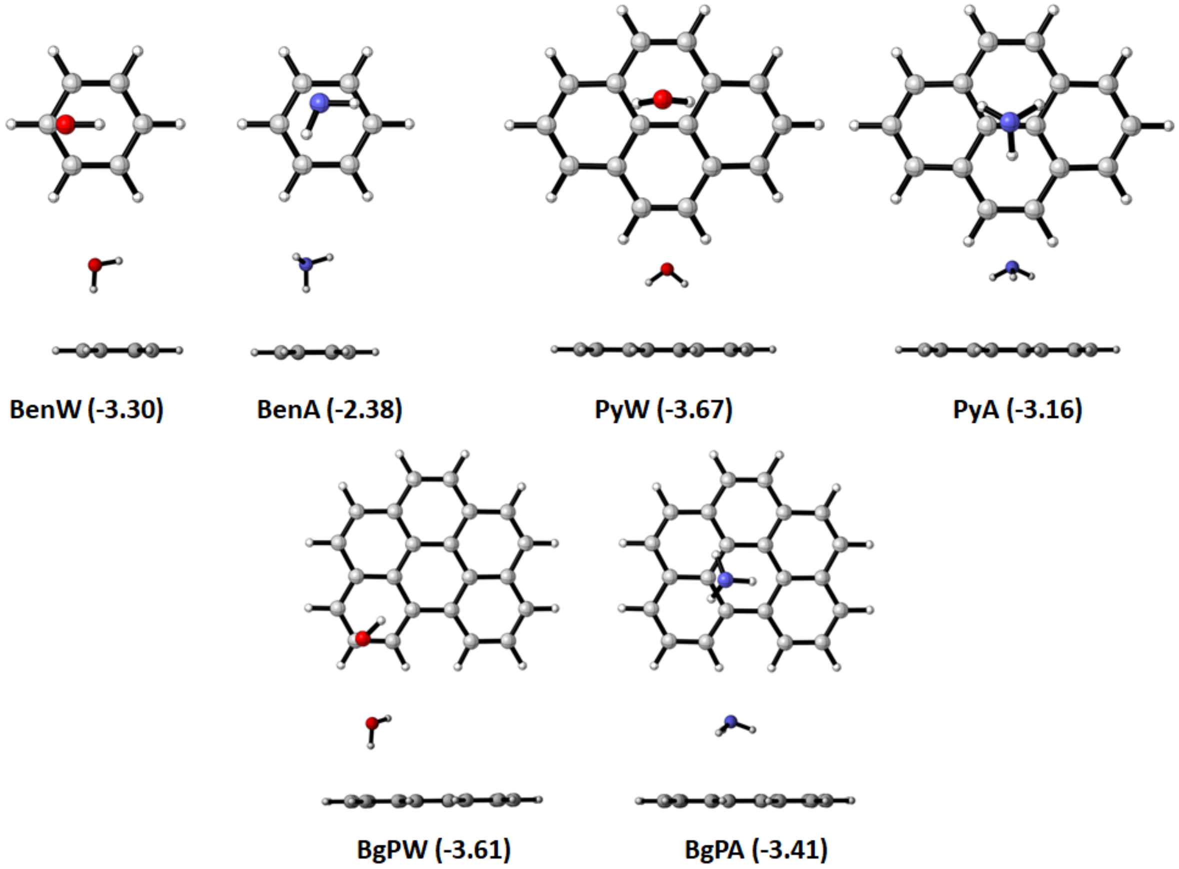

Figure S2 | Optimized PAH–matrix complexes (top-view and side-view) with the calculated bond energy shown in parentheses in kcal mol^{-1}, calculated at the ZORA-BLYP-D3(BJ)/TZ2P level of theory.

Figure S3 | Bond energies of the extra PAH–matrix complexes

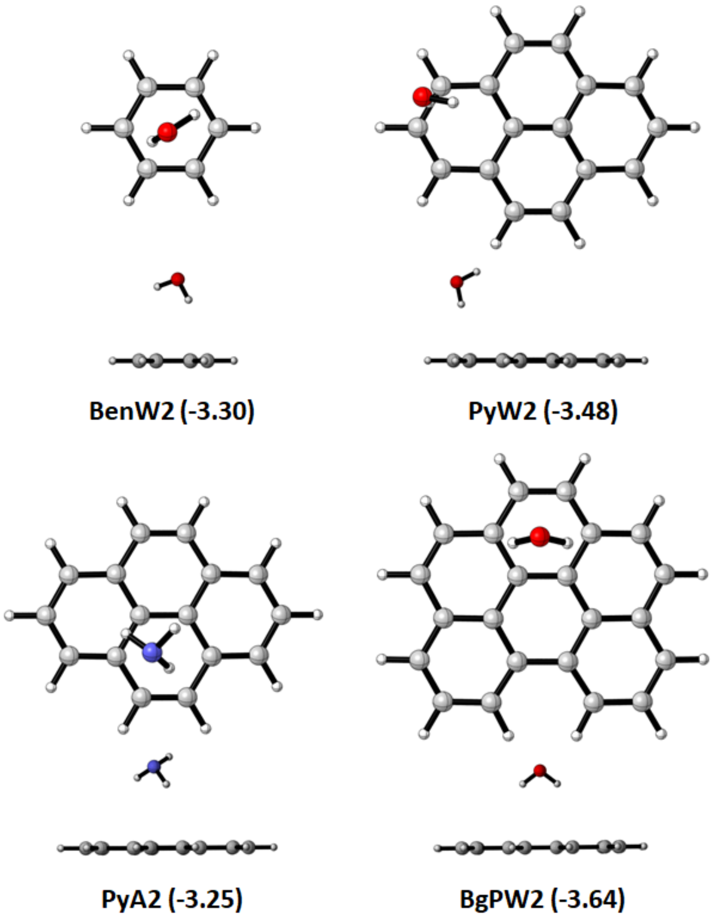

Figure S3 | Extra PAH–matrix complexes (top view and side view) with the calculated bond energy shown in parentheses in kcal mol^{-1}, calculated at the ZORA-BLYP-D3(BJ)/TZ2P level of theory. For these extra PAH–matrix complexes, the calculated charge transfer excitation and corresponding involved molecular orbitals are reported in Table S4 and Figure S11, respectively.

Figure S4 | Orbital interaction diagram of **BenW**

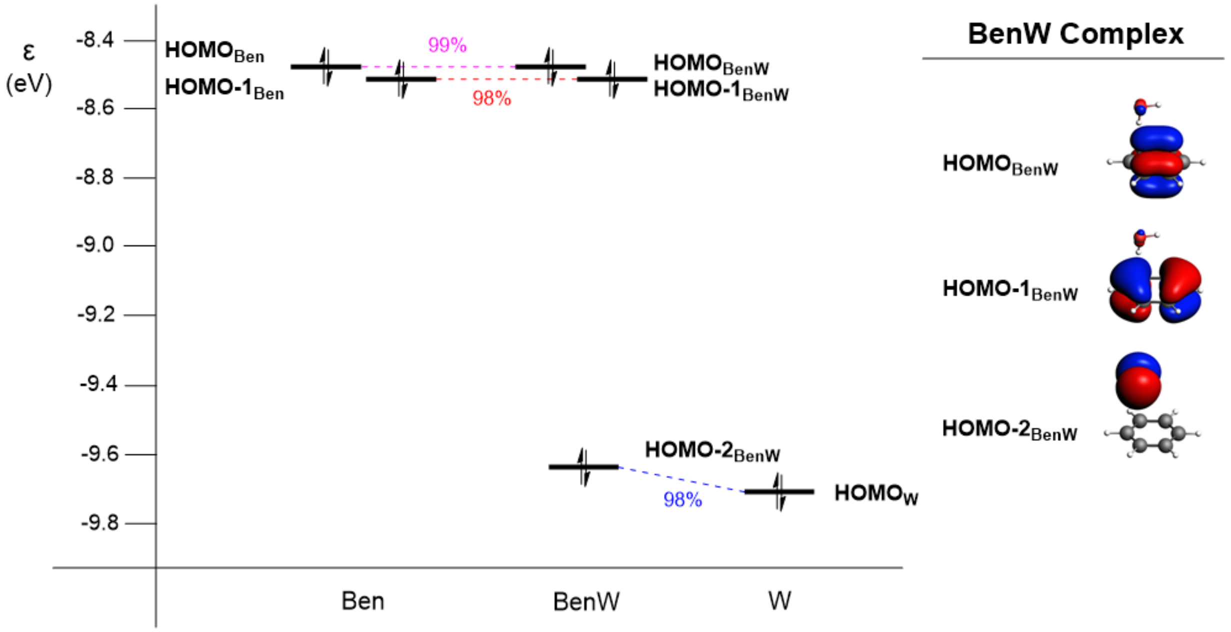

Figure S4 | Orbital interaction diagram of occupied fragment molecular orbitals (FMOs) of **BenW**, with FMO contributions (in %) and visualization of the overall-complex MOs (isosurface at 0.03 au), calculated at ZORA-CAMY-B3LYP/TZ2P level of theory. The FMOs are calculated in the field of the other fragment, as described in Computation Details section 2.2, and reported in Table S3.

Figure S5 | Orbital interaction diagram of **BenA**

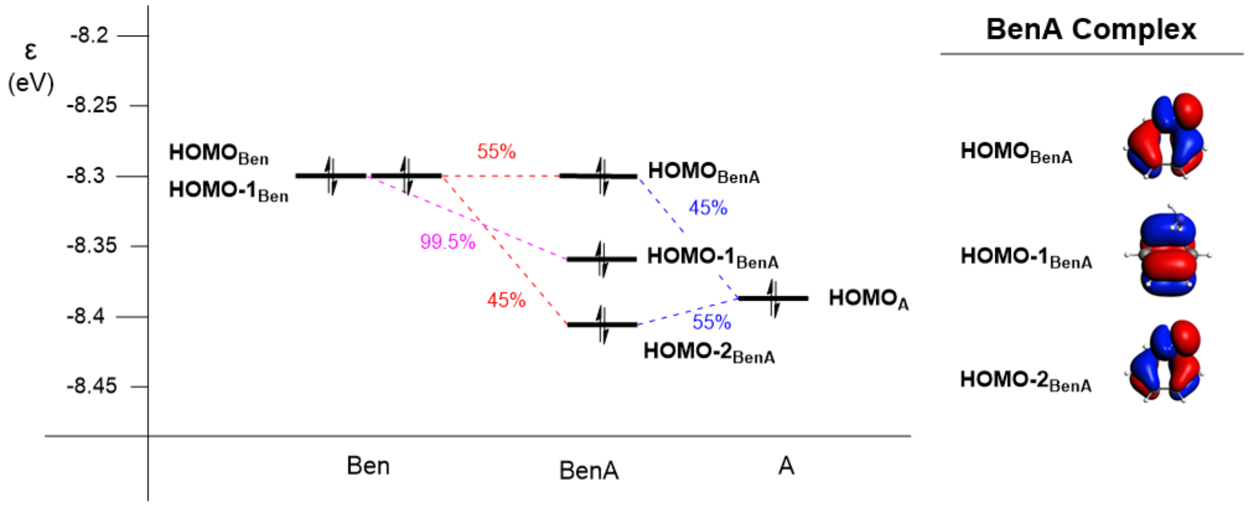

Figure S5 | Orbital interaction diagram of occupied fragment molecular orbitals (FMOs) of **BenA**, with FMO contributions (in %) and visualization of the overall-complex MOs (isosurface at 0.03 au), calculated at ZORA-CAMY-B3LYP/TZ2P level of theory. The FMOs are calculated in the field of the other fragment, as described in Computation Details section 2.2, and reported in Table S3.

Figure S6 | Orbital interaction diagram of **PyW**

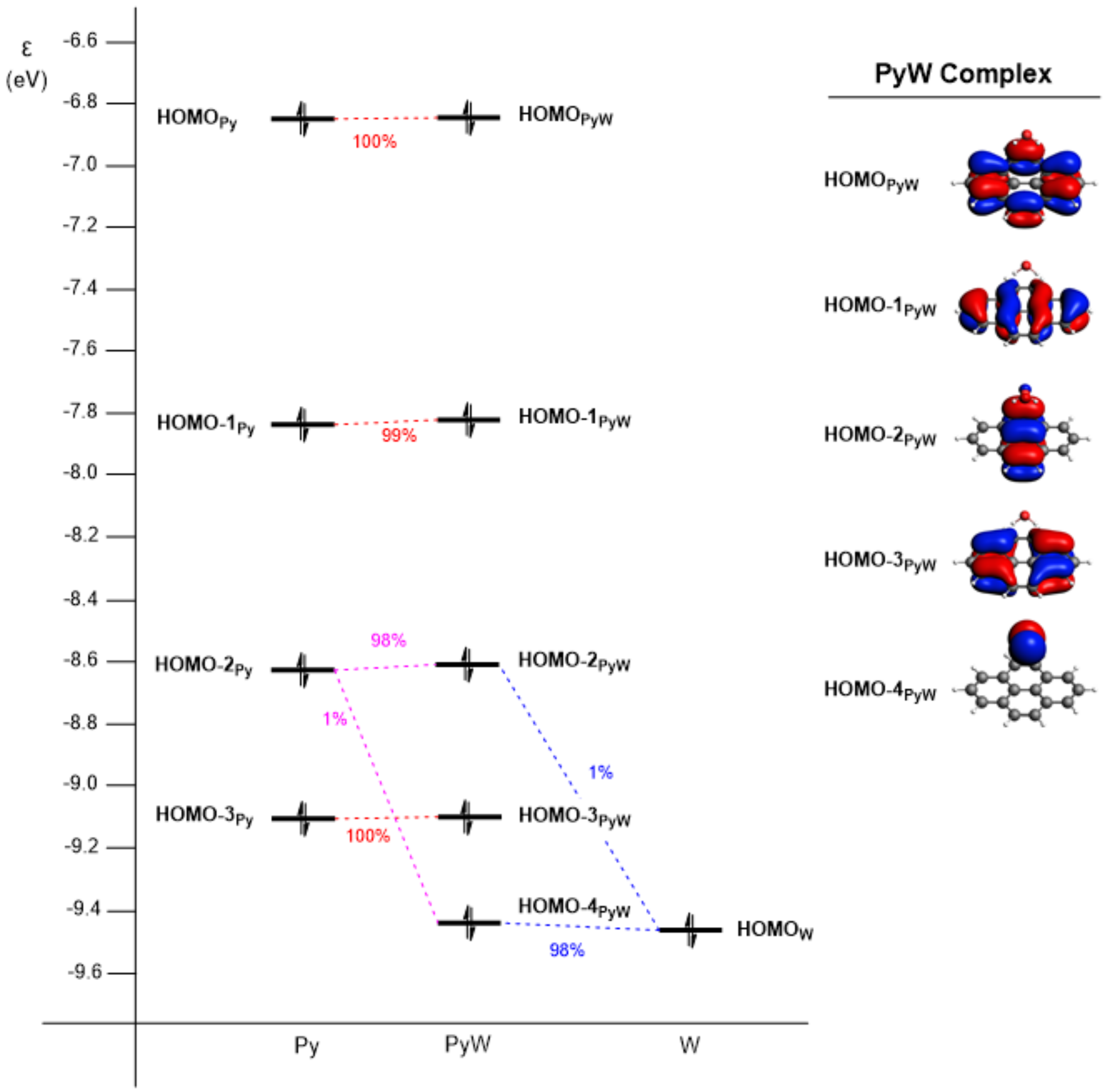

Figure S6 | Orbital interaction diagram of occupied fragment molecular orbitals (FMOs) of **PyW**, with FMO contributions (in %) and visualization of the overall-complex MOs (isosurface at 0.03 au), calculated at ZORA-CAMY-B3LYP/TZ2P level of theory. The FMOs are calculated in the field of the other fragment, as described in Computation Details section 2.2, and reported in Table S3.

Figure S7 | Orbital interaction diagram of **PyA**

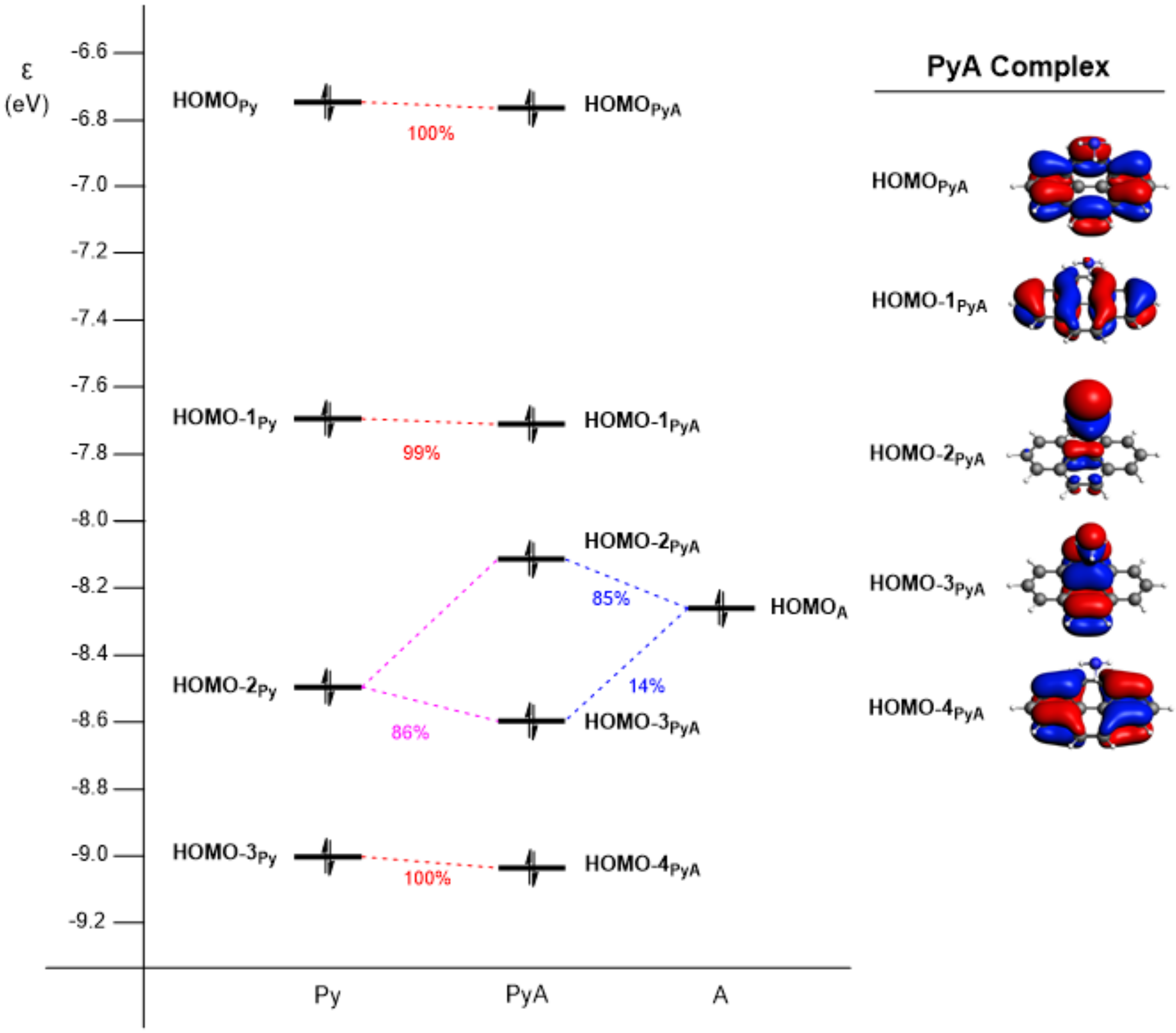

Figure S7 | Orbital interaction diagram of occupied fragment molecular orbitals (FMOs) of **PyA**, with FMO contributions (in %) and visualization of the overall-complex MOs (isosurface at 0.03 au), calculated at ZORA-CAMY-B3LYP/TZ2P level of theory. The FMOs are calculated in the field of the other fragment, as described in Computation Details section 2.2, and reported in Table S3.

Figure S8 | Orbital interaction diagram of **BgPW**

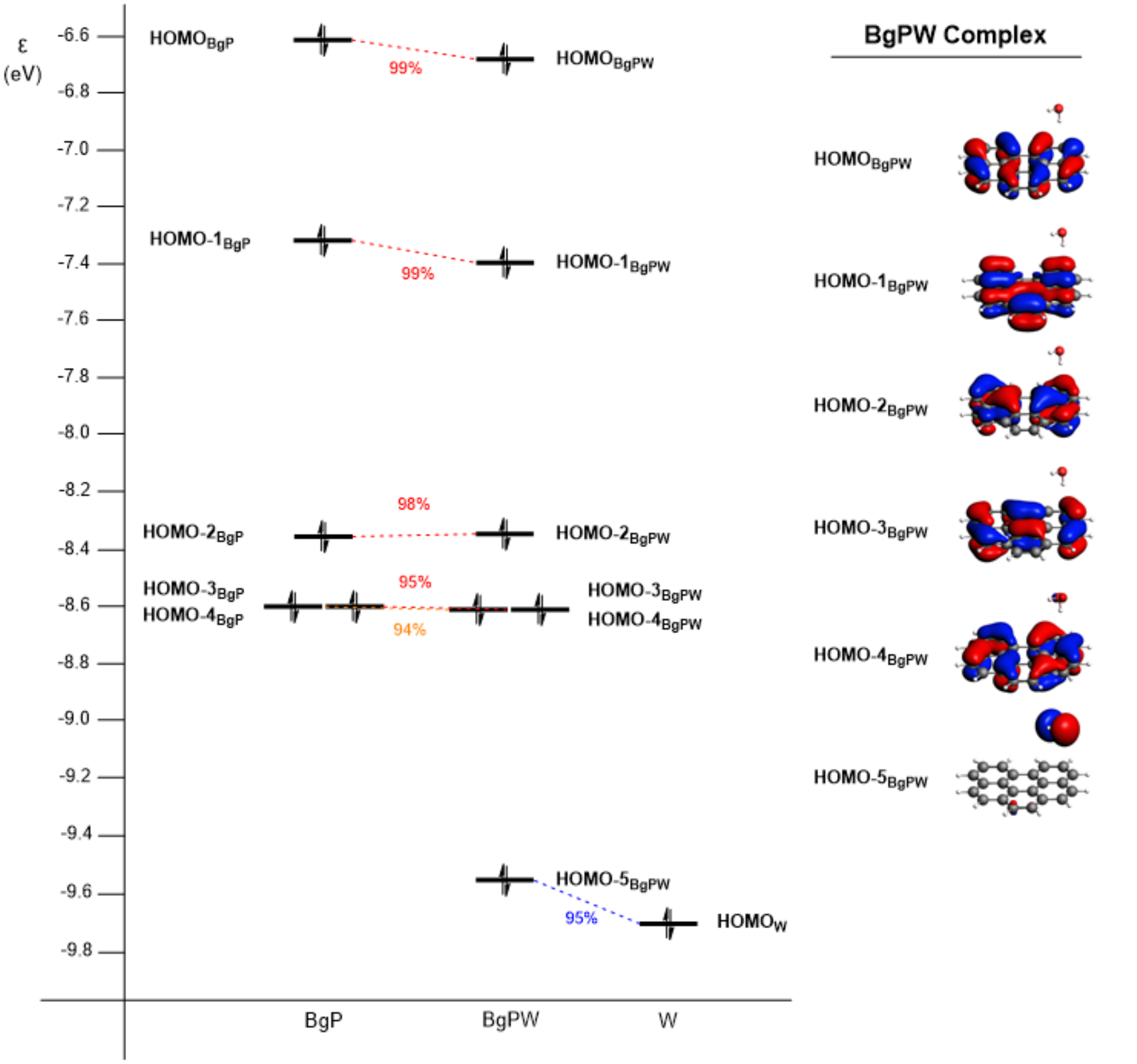

Figure S8 | Orbital interaction diagram of occupied fragment molecular orbitals (FMOs) of **BgPW**, with FMO contributions (in %) and visualization of the overall-complex MOs (isosurface at 0.03 au), calculated at ZORA-CAMY-B3LYP/TZ2P level of theory. The FMOs are calculated in the field of the other fragment, as described in Computation Details section 2.2, and reported in Table S3.

Figure S9 | Orbital interaction diagram of **BgPA**

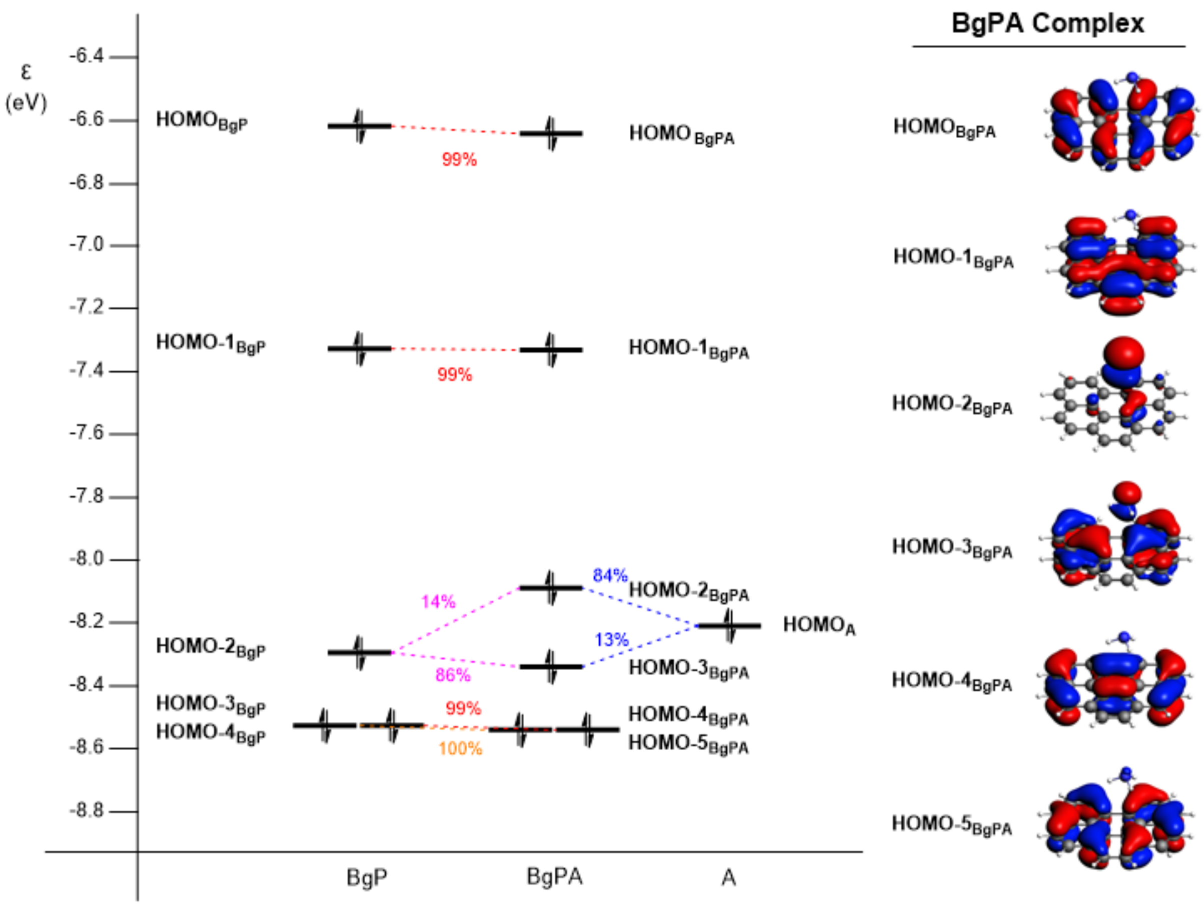

Figure S9 | Orbital interaction diagram of occupied fragment molecular orbitals (FMOs) of **BgPA**, with FMO contributions (in %) and visualization of the overall-complex MOs (isosurface at 0.03 au), calculated at ZORA-CAMY-B3LYP/TZ2P level of theory. The FMOs are calculated in the field of the other fragment, as described in Computation Details section 2.2, and reported in Table S3.

Figure S10 | Overview of molecular orbitals of the PAH–matrix complexes involved in charge transfer excitations.

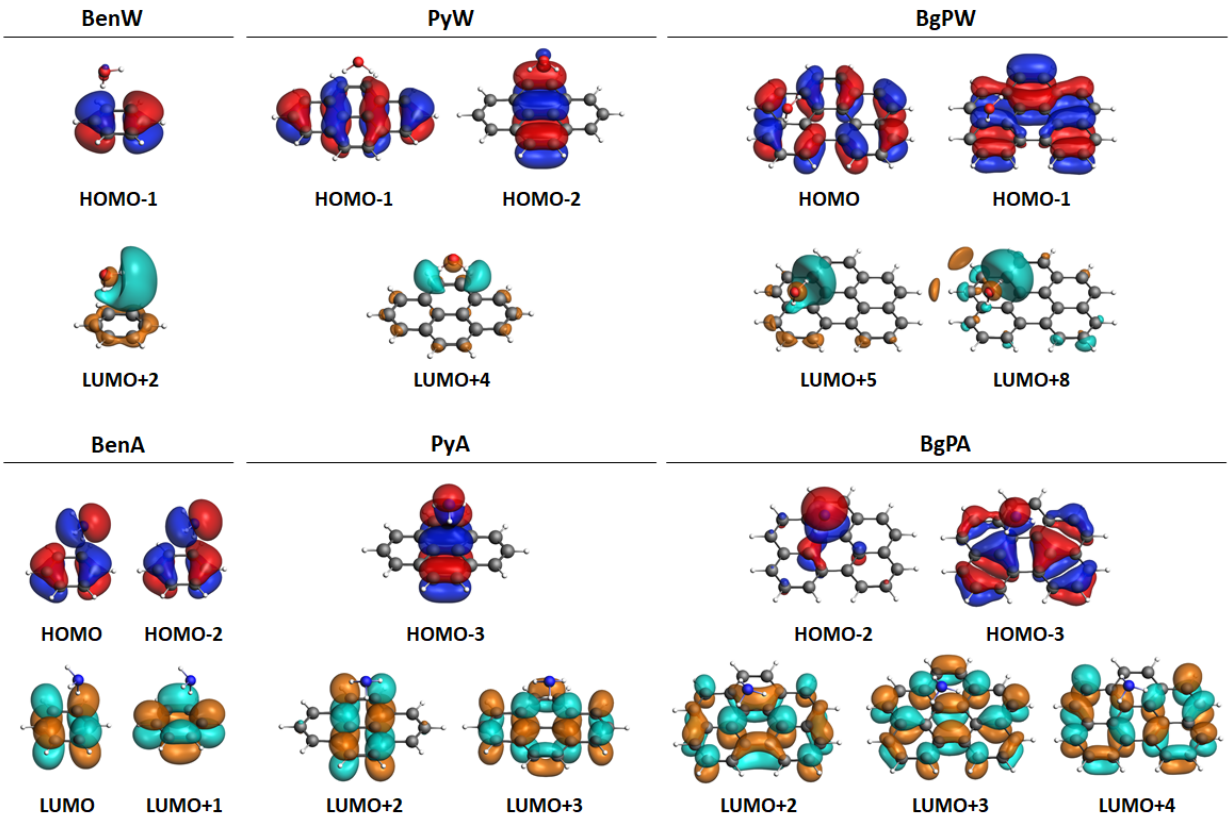

Figure S10 | Overview of all MOs involved in charge transfer excitations for the **BenW**, **BenA**, **PyW**, **PyA**, **BgPW**, and **BgPA** complexes with isosurfaces (at 0.03 au) calculated at ZORA-CAMY-B3LYP/TZ2P level of theory. Occupied MOs are red and blue, while unoccupied MOs are cyan and orange. For clarity, the subscript has not been written out. Optimized structures can be found in Figure 3 of the main text.

Figure S11 | Overview of molecular orbitals of the extra PAH–matrix complexes involved in charge transfer excitations.

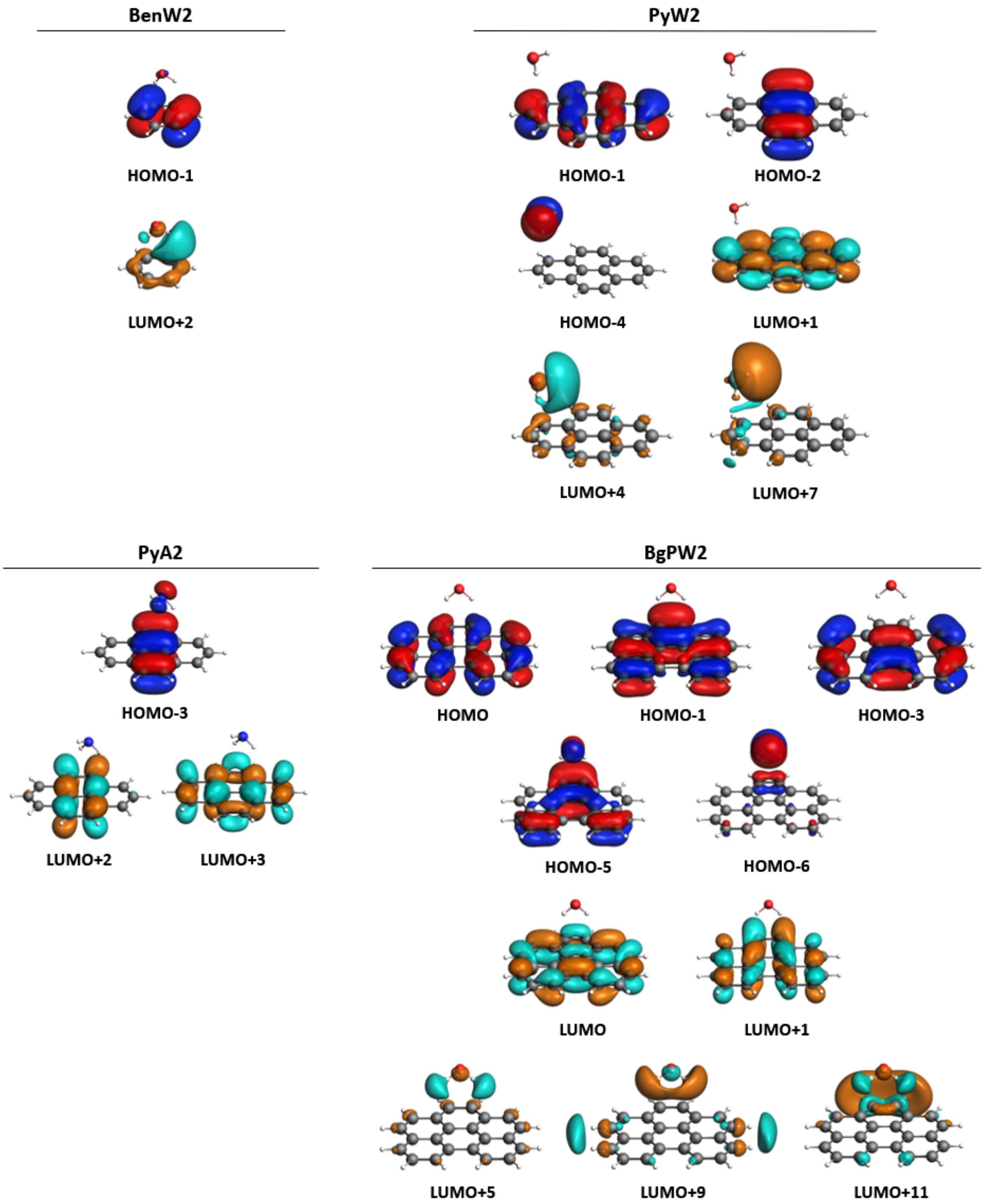

Figure S11 | Overview of all MOs involved in charge transfer excitations for the **BenW2**, **PyA2**, **PyW2**, and **BgPW2** complexes with isosurfaces (at 0.03 au) calculated at ZORA-CAMY-B3LYP/TZ2P level of theory. Occupied MOs are red and blue, while unoccupied MOs are cyan and orange. For clarity, the subscript has not been written out. Optimized structures can be found in Figure S3.

Figure S12 | Molecular orbitals involved in charge transfer excitations in **BenW** and **BenA**

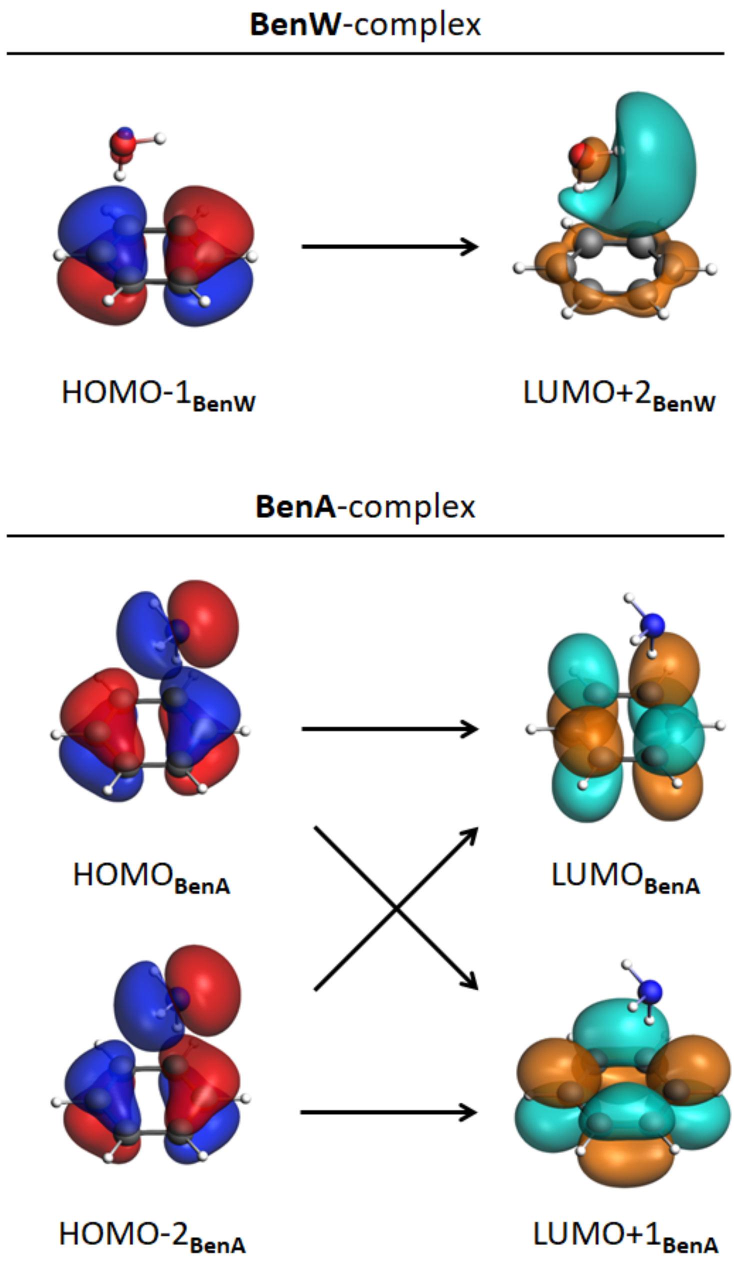

Figure S12 | Relevant MOs involved in charge transfer excitations for **BenW** (top) and **BenA** (bottom) with isosurfaces (at 0.03 au) calculated at CAMY-B3LYP/TZ2P level of theory. Occupied MOs are red and blue while unoccupied MOs are cyan and orange. Optimized structures can be found in Figure 3 of the main text.

Figure S13 | Molecular orbitals involved in charge transfer excitations in BgPW and BgPA

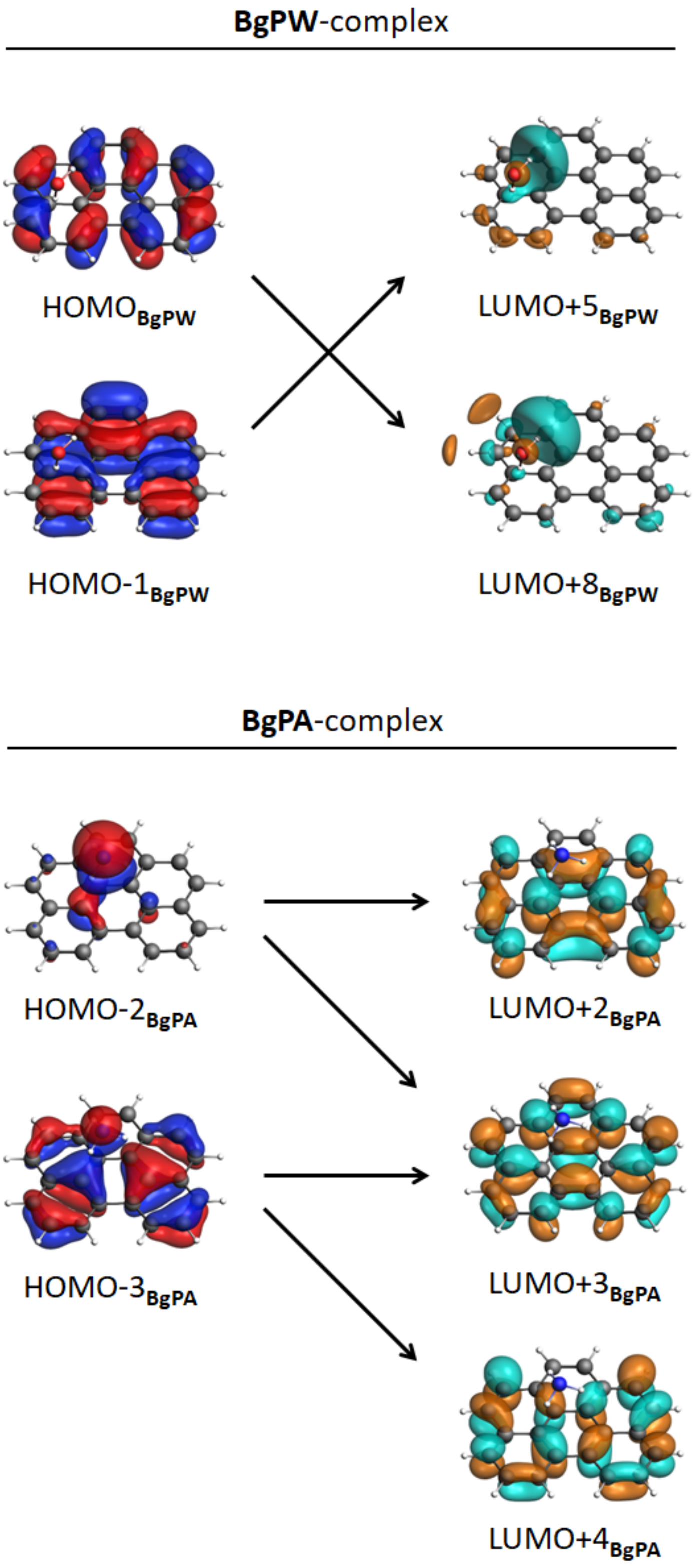

Figure S13 | Relevant MOs involved in charge transfer excitations for **BgPW** (top) and **BgPA** (bottom) with isosurfaces (at 0.03 au) calculated at CAMY-B3LYP/TZ2P level of theory. Occupied MOs are red and blue while unoccupied MOs are cyan and orange. Optimized structures can be found in Figure 3 of the main text.

Supporting Method | Energy decomposition analysis (EDA)

The energy decomposition analysis (EDA) uses Kohn-Sham molecular orbitals (KS MOs) to decompose the bond energy (ΔE_{bond}) of our PAH–matrix complexes into physical meaningful terms.[2] First, the bond energy can be split into the strain energy ΔE_{strain} and interaction energy ΔE_{int}:

$$\Delta E_{bond} = \Delta E_{strain} + \Delta E_{int}$$

where ΔE_{strain} denotes the energy required to deform the optimized monomer fragments to achieve the interacting system, and ΔE_{int} accounts for the actual stabilizing interaction between the monomers. The interaction energy can be further decomposed into four terms: electrostatic interaction ΔV_{elstat}, Pauli repulsion ΔE_{Pauli}, orbital interaction $\Delta E_{oi,}$ and dispersion correction ΔE_{disp}:

$$\Delta E_{int} = \Delta V_{elstat} + \Delta E_{Pauli} + \Delta E_{oi} + \Delta E_{disp}$$

The electrostatic interaction ΔV_{elstat} describes the classical Coulomb interaction between the charge distributions of the deformed monomers and is usually attractive for neutral systems. The Pauli repulsion ΔE_{Pauli} describes the destabilizing interaction between the overlapping occupied orbitals of the monomers and is responsible for steric repulsion. The orbital energy ΔE_{oi} describes the donor-acceptor interactions of occupied orbitals of one monomer and the unoccupied orbitals of the other monomer, such as the HOMO–LUMO interactions. It also describes polarization in monomers (*i.e.* the mixing of empty and occupied orbitals of a monomer as a result of the presence of the other monomer). Lastly, the dispersion correction ΔE_{disp} accounts for the dispersion interactions.

All EDA calculations were performed with the Amsterdam Density Functional (ADF) program, version 2019.102.

[2] **(a)** Bickelhaupt, F. M.; Baerends, E. J. in *Reviews in Computational Chemistry*, ed. K. B. Lipkowitz and D. B. Boyd, Wiley, Hoboken, 2000; **(b)** Zhao, L.; von Hopffgarten,M.; Andrada, D. M. and Frenking, G. *WIREs Comput. Mol. Sci.* **2018**, *8*, e1345; **(c)** Hamlin, T. A.; Vermeeren, P.; Fonseca Guerra C.; Bickelhaupt, F. M. in *Complementary Bonding Analyses*, ed. S. Grabowsky, De Gruyter, Berlin, 2021.

Table S1 | Energy decomposition analysis of the PAH–matrix complexes

Table S1 | Energy Decomposition Analysis (EDA) of the PAH–matrix complexes, calculated at the ZORA-CAMY-B3LYP-D3(BJ)/TZ2P level of theory. All energies are displayed in kcal mol^{-1}.

Complexes	ΔE_{bond}	$\Delta E_{\mathrm{strain}}$	ΔE_{int}	$\Delta V_{\mathrm{elstat}}$	$\Delta E_{\mathrm{Pauli}}$	ΔE_{oi}	ΔE_{disp}
**BenW**	–3.27	0.40	–3.67	–2.63	1.97	–1.72	–1.29
**BenA**	–2.27	0.36	–2.63	–1.59	1.24	–0.93	–1.35
**PyW**	–3.16	0.75	–3.91	–2.04	1.36	–1.21	–2.02
**PyA**	–2.57	0.79	–3.36	–2.07	1.84	–0.68	–2.45
**BgPW**	–3.02	0.86	–3.88	–2.67	2.19	–1.87	–1.53
**BgPA**	–2.66	0.89	–3.55	–2.14	1.96	–0.73	–2.64

Table S2 | Orbital overlap in the PAH–matrix complexes

Table S2 | Overlap between HOMO of the matrix (ammonia or water) and the benzene or PAH MO involved in the mixing occurring in the PAH–matrix complexes, calculated at ZORA-CAMY-B3LYP/TZ2P level of theory.

**Complex**	**Water (HOMO)** $| HOMO_{\mathbf{W}}\rangle$	**Ammonia (HOMO)** $| HOMO_{\mathbf{A}}\rangle$
**Benzene (HOMO)** $\langle HOMO_{\mathbf{Ben}} |$	0.0000	0.0166
**Pyrene (HOMO-2)** $\langle HOMO\text{-}2_{\mathbf{Py}} |$	0.0175	0.0266
**Benzo[ghi]perylene (HOMO-2)** $\langle HOMO\text{-}2_{\mathbf{BgP}} |$	0.0015	0.0141

Table S3 | Fock Matrix Symmetrized Fragment Orbital (FMATSFO)

While the mixing of the water molecule with benzene and other PAHs is minimal for all complexes, the orbitals belonging mostly to water (HOMO-$2_{\mathbf{BenW}}$, HOMO-$4_{\mathbf{PyW}}$, and HOMO-$5_{\mathbf{BgPW}}$) raise in energy relative to the isolated water molecule when in the PAH–water complex. This destabilization of the water MO can be attributed to the fact that the water molecule is placed in the field of the aromatic molecules and experiences electronic repulsion, pushing the energy level up. Similarly, the electron density in the occupied MOs (mostly) localized on the PAH of the PAH–water complex is stabilized due to the presence of the electropositive hydrogen atoms of water and are therefore lowered in energy. Similar results are found for the PAH–ammonia complexes. More details on the computational method of the FMATSFO method can be found in section 2.2 of the main text.

Table S3 | Molecular orbital energies of the PAH–matrix fragment calculated isolated (original energy) and when calculated in the field of the other fragment (FMATSFO energy), calculated at ZORA-CAMY-B3LYP/TZ2P level of theory.

System	MO of Fragment	Original energy (eV)	FMATSFO energy (eV)
**BenW**	$HOMO_W$	-10.2	-9.69
	$HOMO_{Ben}$	-8.16	-8.54
	HOMO-1_{Ben}	-8.16	-8.47
**BenA**	$HOMO_A$	-8.84	-8.38
	HOMO-1_{Ben}	-8.16	-8.31
	$HOMO_{Ben}$	-8.16	-8.30
**PyW**	$HOMO_W$	-10.2	-9.48
	$HOMO_{Py}$	-6.63	-6.84
	HOMO-1_{Py}	-7.57	-7.83
	HOMO-2_{Py}	-8.38	-8.64
	HOMO-2_{Py}	-8.89	-9.14
**PyA**	$HOMO_A$	-8.90	-8.24
	$HOMO_{Py}$	-6.63	-6.75
	HOMO-1_{Py}	-7.58	-7.69
	HOMO-2_{Py}	-8.38	-8.51
	HOMO-3_{Py}	-8.89	-9.01
**BgPW**	$HOMO_W$	-10.2	-9.69
	$HOMO_{BgP}$	-6.50	-6.67
	HOMO-1_{BgP}	-7.22	-7.38
	HOMO-2_{BgP}	-8.18	-8.38
	HOMO-3_{BgP}	-8.41	-8.58
	HOMO-4_{BgP}	-8.44	-8.60
**BgPA**	$HOMO_A$	-8.89	-8.21
	$HOMO_{BgP}$	-6.50	-6.61
	HOMO-1_{BgP}	-7.22	-7.33
	HOMO-2_{BgP}	-8.18	-8.31
	HOMO-3_{BgP}	-8.41	-8.52
	HOMO-4_{BgP}	-8.44	-8.55

Table S4 | TDDFT results of the extra PAH–matrix complexes

Table S4 | Charge transfer excitations in the benzene/PAHs–matrix complexes, calculated at ZORA-CAMY-B3LYP/TZ2P level of theory.[a]

**Complexes**	**Transition (eV)**	**Oscillator strength**	**Weight**	**From MO (complex)**	**To MO (complex)**	**Character of charge transfer direction**
**BenW2**	7.09	0.245	48.0%	HOMO-1$_{\mathbf{BenW2}}$	LUMO+2$_{\mathbf{BenW2}}$	$\pi_{PAH} \rightarrow \sigma^*_{PAH} + \sigma^*_{H_2O}$
**PyW2**	8.37	0.016	25.1%	HOMO-2$_{\mathbf{PyW2}}$	LUMO+4$_{\mathbf{PyW2}}$	$\pi_{PAH} \rightarrow \sigma^*_{PAH} + \sigma^*_{H_2O}$
	7.54	0.033	23.0%	HOMO-2$_{\mathbf{PyW2}}$	LUMO+4$_{\mathbf{PyW2}}$	$\pi_{PAH} \rightarrow \sigma^*_{PAH} + \sigma^*_{H_2O}$
			30.7%	HOMO-1$_{\mathbf{PyW2}}$	LUMO+7$_{\mathbf{PyW2}}$	$\pi_{PAH} \rightarrow \sigma^*_{PAH} + \sigma^*_{H_2O}$
	7.58	0.024	30.0%	HOMO-2$_{\mathbf{PyW2}}$	LUMO+4$_{\mathbf{PyW2}}$	$\pi_{PAH} \rightarrow \sigma^*_{PAH} + \sigma^*_{H_2O}$
			24.6%	HOMO-1$_{\mathbf{PyW2}}$	LUMO+7$_{\mathbf{PyW2}}$	$\pi_{PAH} \rightarrow \sigma^*_{PAH} + \sigma^*_{H_2O}$
	7.38	0.245	34.1%	HOMO-4$_{\mathbf{PyW2}}$	LUMO+1$_{\mathbf{PyW2}}$	$LP_{H_2O} \rightarrow \pi^*_{PAH}$
	7.30	0.213	57.8%	HOMO-4$_{\mathbf{PyW2}}$	LUMO+1$_{\mathbf{PyW2}}$	$LP_{H_2O} \rightarrow \pi^*_{PAH}$
	6.74	0.027	70.9%	HOMO-1$_{\mathbf{PyW2}}$	LUMO+4$_{\mathbf{PyW2}}$	$\pi_{PAH} \rightarrow \sigma^*_{PAH} + \sigma^*_{H_2O}$
**PyA2**	7.28	0.804	56.8%	HOMO-3$_{\mathbf{PyA2}}$	LUMO+3$_{\mathbf{PyA2}}$	π_{PAH} & $LP_{NH_3} \rightarrow \pi^*_{PAH}$
	6.73	0.715	82.4%	HOMO-3$_{\mathbf{PyA2}}$	LUMO+2$_{\mathbf{PyA2}}$	π_{PAH} & $LP_{NH_3} \rightarrow \pi^*_{PAH}$
**BgPW2**	7.93	0.035	10.4%	HOMO-3$_{\mathbf{BgPW2}}$	LUMO+5$_{\mathbf{BgPW2}}$	$\pi_{PAH} \rightarrow \sigma^*_{PAH} + \sigma^*_{H_2O}$
	7.35	0.055	27.5%	HOMO-1$_{\mathbf{BgPW2}}$	LUMO+9$_{\mathbf{BgPW2}}$	$\pi_{PAH} \rightarrow \sigma^*_{PAH} + \sigma^*_{H_2O}$
	7.31	0.069	10.1%	HOMO-1$_{\mathbf{BgPW2}}$	LUMO+9$_{\mathbf{BgPW2}}$	$\pi_{PAH} \rightarrow \sigma^*_{PAH} + \sigma^*_{H_2O}$
			17.1%	HOMO-1$_{\mathbf{BgPW2}}$	LUMO+11$_{\mathbf{BgPW2}}$	$\pi_{PAH} \rightarrow \sigma^*_{PAH} + \sigma^*_{H_2O}$
	6.67	0.013	88.8%	HOMO$_{\mathbf{BgPW2}}$	LUMO+11$_{\mathbf{BgPW2}}$	$\pi_{PAH} \rightarrow \sigma^*_{PAH} + \sigma^*_{H_2O}$
	6.53	1.083	5.4%	HOMO-5$_{\mathbf{BgPW2}}$	LUMO+1$_{\mathbf{BgPW2}}$	π_{PAH} & $LP_{H_2O} \rightarrow \pi^*_{PAH}$
			3.2%	HOMO-6$_{\mathbf{BgPW2}}$	LUMO+1$_{\mathbf{BgPW2}}$	π_{PAH} & $LP_{H_2O} \rightarrow \pi^*_{PAH}$
	6.48	0.043	85.1%	HOMO$_{\mathbf{BgPW2}}$	LUMO+9$_{\mathbf{BgPW2}}$	$\pi_{PAH} \rightarrow \sigma^*_{PAH} + \sigma^*_{H_2O}$
	5.95	0.268	20.4%	HOMO-5$_{\mathbf{BgPW2}}$	LUMO$_{\mathbf{BgPW2}}$	π_{PAH} & $LP_{H_2O} \rightarrow \pi^*_{PAH}$
			10.1%	HOMO-6$_{\mathbf{BgPW2}}$	LUMO$_{\mathbf{BgPW2}}$	π_{PAH} & $LP_{H_2O} \rightarrow \pi^*_{PAH}$

[a] Lone pairs are abbreviated as LP. The MOs involved in the charge transfer excitations (columns 5 and 6) can be found in Figure S11**.**

Supporting Data | Cartesian coordinates of the optimized PAH–matrix complexes

Cartesian coordinates (in Å) and total bond energies (E_{bond}, in kcal mol^{-1}) of all optimized complexes, calculated at ZORA-BLYP-D3(BJ)/TZ2P level of theory in the gas phase. The number of imaginary frequencies (NIMAG) is 0 for each complex.

Complex BenA E_{bond} = -2118.21 kcal mol^{-1} NIMAG: 0

1	C	0.007269	1.432563	0.405154
2	C	-1.183345	0.708194	0.272432
3	C	-1.147732	-0.689353	0.196093
4	C	0.078860	-1.362049	0.252388
5	C	1.269271	-0.637360	0.385197
6	C	1.233466	0.759817	0.461734
7	H	-2.135905	1.230428	0.226787
8	H	-2.071856	-1.251693	0.088720
9	H	0.106343	-2.447318	0.191536
10	H	2.221846	-1.160065	0.429318
11	H	2.158172	1.322610	0.564966
12	H	-0.020662	2.518018	0.464795
13	N	-0.232769	-0.040095	-3.374531
14	H	-0.310800	-0.109119	-2.356570
15	H	-0.128450	0.954682	-3.585319
16	H	0.656292	-0.479260	-3.622700

Complex BenW E_{bond} = -2002.37 kcal mol^{-1} NIMAG: 0

1	C	0.093551	1.288670	0.000000
2	C	0.162197	0.591582	1.213257
3	C	0.299725	-0.801438	1.212752
4	C	0.368792	-1.497445	0.000000
5	C	0.299725	-0.801438	-1.212752
6	C	0.162197	0.591582	-1.213257
7	H	0.105169	1.132564	2.154396
8	H	0.352424	-1.342420	2.154421
9	H	0.477160	-2.579546	0.000000
10	H	0.352424	-1.342420	-2.154421
11	H	0.105169	1.132564	-2.154396
12	H	-0.012351	2.370742	0.000000
13	O	-3.286053	0.451551	0.000000
14	H	-3.357227	-0.518129	0.000000
15	H	-2.322901	0.609529	0.000000

Complex PyA E_{bond} = -4446.49 kcal mol^{-1} NIMAG: 0

1	C	-1.578498	-0.283608	0.077076
2	C	-2.810680	-1.012938	0.063757
3	C	-2.759010	-2.449243	0.055750
4	C	-1.568393	-3.117243	0.059803
5	C	2.121243	-2.359600	0.070973
6	C	2.111504	-0.963702	0.077462
7	C	0.896950	-0.252230	0.081355
8	C	-0.335128	-0.981370	0.081650
9	C	-0.315699	-2.412936	0.071916
10	C	0.924620	-3.078724	0.067392
11	C	-0.345349	1.852300	0.072321
12	H	-3.697248	-2.998997	0.046359
13	H	-1.548709	-4.204549	0.053405
14	H	3.068817	-2.891264	0.068262
15	H	3.048222	-0.411125	0.078408
16	C	0.845388	1.184063	0.076539
17	H	0.941106	-4.166157	0.061159
18	C	-1.598185	1.148055	0.072990
19	C	-4.025189	-0.301093	0.054775
20	C	-4.034829	1.094900	0.056700
21	C	-2.838359	1.813949	0.064585
22	H	-2.854806	2.901394	0.063377
23	H	1.783676	1.733861	0.076730
24	H	-0.364975	2.939603	0.069202
25	H	-4.982361	1.626626	0.050851
26	H	-4.961927	-0.853572	0.046485
27	N	-0.749617	-0.679912	-3.421121
28	H	-1.688003	-0.677158	-3.012963
29	H	-0.260645	0.102419	-2.978417
30	H	-0.297115	-1.522251	-3.056781

Complex PyW E_{bond} = -4330.22 kcal mol⁻¹ NIMAG: 0

1	C	-1.571054	-0.215356	0.171417
2	C	-2.807997	-0.937737	0.147632
3	C	-2.764999	-2.374355	0.131839
4	C	-1.579262	-3.049737	0.132153
5	C	2.115698	-2.315188	0.158015
6	C	2.115077	-0.919380	0.185363
7	C	0.905543	-0.199549	0.191947
8	C	-0.331288	-0.921345	0.172133
9	C	-0.321701	-2.353831	0.148143
10	C	0.915016	-3.027143	0.139379
11	C	-0.323611	1.912364	0.216152
12	H	-3.706362	-2.918084	0.114778
13	H	-1.566612	-4.136747	0.114802
14	H	3.059885	-2.852752	0.152111
15	H	3.055378	-0.373334	0.200641
16	C	0.862512	1.236838	0.216272
17	H	0.924622	-4.114294	0.120374
18	C	-1.581072	1.216724	0.191571
19	C	-4.017956	-0.217553	0.140716
20	C	-4.018078	1.178266	0.160148
21	C	-2.817220	1.889844	0.186330
22	H	-2.827188	2.977160	0.202382
23	H	1.804144	1.780394	0.233402
24	H	-0.336437	2.999537	0.233417
25	H	-4.962103	1.716126	0.155787
26	H	-4.958052	-0.763778	0.122336
27	O	-1.235058	-1.367973	-3.267641
28	H	-0.543398	-1.657803	-2.646316
29	H	-1.845206	-0.866095	-2.698192

Complex BgPA E_{bond} = -5813.85 kcal mol⁻¹ NIMAG: 0

1	C	-3.506313	0.049393	1.165268
2	C	-3.532376	-0.020247	-1.315241
3	C	-2.815250	0.063721	2.342857
4	C	-2.825766	-0.004126	-0.096207
5	C	-2.847033	-0.074305	-2.522178
6	C	-1.383714	0.024541	2.360512
7	C	-1.392574	-0.044055	-0.114171
8	C	-1.448915	-0.112501	-2.544551
9	C	-0.676371	-0.031313	1.125442
10	C	-0.694464	-0.098457	-1.361392
11	C	0.751607	-0.073665	1.127287
12	C	0.772503	-0.137460	-1.359251
13	C	1.457376	-0.054086	2.364159
14	C	1.469109	-0.126928	-0.110209
15	C	1.528303	-0.176566	-2.540909
16	C	2.889163	-0.089234	2.350215
17	C	2.901919	-0.156652	-0.088809
18	C	2.926753	-0.205880	-2.515273
19	C	3.581433	-0.138367	1.174256
20	C	3.610493	-0.196899	-1.306357
21	H	-4.593183	0.078029	1.163117
22	H	-4.619224	0.008883	-1.297836
23	H	-3.344156	0.104434	3.292039
24	H	-3.397218	-0.087946	-3.458998
25	C	-0.652132	0.039157	3.576752
26	H	-0.949403	-0.155928	-3.505738
27	C	0.724479	0.001813	3.578570
28	H	1.029639	-0.183353	-3.503438
29	H	3.417162	-0.075353	3.300741
30	H	3.478108	-0.235629	-3.450994
31	H	4.668335	-0.163516	1.174620
32	H	4.697397	-0.219192	-1.286150
33	H	-1.200136	0.080702	4.514892
34	H	1.271476	0.014315	4.518129
35	N	0.744002	3.372795	-0.192084
36	H	1.095095	3.058221	0.715799
37	H	-0.220377	3.035203	-0.245050
38	H	1.263152	2.831207	-0.887549

Complex BgPW E_{bond} = -5697.33 kcal mol^{-1} NIMAG: 0

1	C	-3.632363	-0.161616	1.328431
2	C	-3.653126	-0.064965	-1.151339
3	C	-2.943670	-0.197795	2.507016
4	C	-2.949085	-0.103244	0.068670
5	C	-2.965376	-0.007730	-2.356800
6	C	-1.511837	-0.177981	2.527235
7	C	-1.515710	-0.083271	0.053124
8	C	-1.566910	0.011344	-2.376934
9	C	-0.801881	-0.120413	1.293667
10	C	-0.815183	-0.026510	-1.192639
11	C	0.626732	-0.099260	1.298631
12	C	0.652497	-0.010310	-1.188040
13	C	1.330441	-0.135376	2.536400
14	C	1.347552	-0.044246	0.062447
15	C	1.410874	0.037541	-2.367990
16	C	2.762229	-0.112770	2.525121
17	C	2.781164	-0.024401	0.087478
18	C	2.810450	0.055970	-2.339035
19	C	3.458000	-0.058473	1.351461
20	C	3.492482	0.027621	-1.128139
21	H	-4.719459	-0.176490	1.324251
22	H	-4.740317	-0.080184	-1.136011
23	H	-3.474688	-0.241954	3.454885
24	H	-3.513849	0.022826	-3.294180
25	C	-0.782167	-0.213992	3.744254
26	H	-1.065369	0.057395	-3.336938
27	C	0.594768	-0.193231	3.748943
28	H	0.915383	0.066130	-3.331330
29	H	3.288099	-0.139831	3.476496
30	H	3.363686	0.094165	-3.273112
31	H	4.544921	-0.040992	1.354277
32	H	4.579110	0.046221	-1.106559
33	H	-1.332185	-0.258370	4.681132
34	H	1.139806	-0.221240	4.689276
35	O	2.437490	3.454657	-1.990392
36	H	2.522670	2.484155	-1.942940
37	H	1.768799	3.658869	-1.314210

Complex BenW2 E_{bond} = -2002.08 kcal mol^{-1} NIMAG: 0

1	C	0.411622	0.544553	1.663153
2	C	-0.885503	0.935721	1.309813
3	C	-1.078982	2.048228	0.483562
4	C	0.023515	2.770362	0.010887
5	C	1.320079	2.380436	0.364252
6	C	1.514448	1.267037	1.190344
7	H	-1.741066	0.371422	1.672538
8	H	-2.086408	2.351233	0.207651
9	H	-0.127616	3.634741	-0.631733
10	H	2.176586	2.937795	-0.006898
11	H	2.521810	0.962030	1.463756
12	H	0.562979	-0.320842	2.304152
13	O	0.465188	-0.452106	-1.743894
14	H	-0.081113	0.317701	-1.977307
15	H	0.714480	-0.273850	-0.819086

Complex PyA2 E_{bond} = -4446.60 kcal mol⁻¹ NIMAG: 0

1	C	-1.570301	-0.380181	-0.011766
2	C	-2.804370	-1.103949	0.050964
3	C	-2.756512	-2.535801	0.166232
4	C	-1.567669	-3.205349	0.212845
5	C	2.123938	-2.461683	0.121547
6	C	2.118097	-1.070266	0.009176
7	C	0.905381	-0.356943	-0.036006
8	C	-0.328686	-1.079909	0.034300
9	C	-0.313021	-2.507279	0.147383
10	C	0.925636	-3.174606	0.189915
11	C	-0.332018	1.745223	-0.193571
12	H	-3.696304	-3.080719	0.216416
13	H	-1.550708	-4.289207	0.300769
14	H	3.070116	-2.994822	0.156495
15	H	3.056118	-0.522595	-0.043217
16	C	0.857004	1.075077	-0.151420
17	H	0.939557	-4.258587	0.277097
18	C	-1.586271	1.047198	-0.126781
19	C	-4.016741	-0.390672	-0.003919
20	C	-4.022711	1.000753	-0.118166
21	C	-2.824492	1.714382	-0.179242
22	H	-2.837898	2.797944	-0.269659
23	H	1.796349	1.620362	-0.204697
24	H	-0.348955	2.828558	-0.284187
25	H	-4.968900	1.533292	-0.159473
26	H	-4.955084	-0.938453	0.044266
27	N	-0.793215	0.367586	-3.581125
28	H	-1.700204	0.268736	-3.118319
29	H	-0.121851	0.531338	-2.826879
30	H	-0.569480	-0.554928	-3.960957

Complex PyW2 E_{bond} = -4330.08 kcal mol⁻¹ NIMAG: 0

1	C	-1.506387	-0.326945	0.172253
2	C	-2.737402	-1.056670	0.226940
3	C	-2.686479	-2.492592	0.239565
4	C	-1.496120	-3.160486	0.205204
5	C	2.191299	-2.404213	0.071076
6	C	2.181696	-1.008236	0.052460
7	C	0.968010	-0.296107	0.083836
8	C	-0.263237	-1.025304	0.135767
9	C	-0.244099	-2.456984	0.153559
10	C	0.995748	-3.122997	0.120589
11	C	-0.273414	1.809302	0.100923
12	H	-3.624245	-3.041528	0.277509
13	H	-1.476418	-4.247739	0.216813
14	H	3.138456	-2.936196	0.047306
15	H	3.118022	-0.456284	0.014313
16	C	0.916000	1.140200	0.066965
17	H	1.012525	-4.210337	0.134952
18	C	-1.525732	1.105623	0.156203
19	C	-3.951982	-0.343746	0.266388
20	C	-3.960829	1.053506	0.251853
21	C	-2.764501	1.772153	0.195781
22	H	-2.782098	2.859188	0.180760
23	H	1.853797	1.689378	0.027109
24	H	-0.293097	2.896433	0.088115
25	H	-4.907558	1.585308	0.281433
26	H	-4.888372	-0.894537	0.308544
27	O	-4.382537	0.224728	-3.081247
28	H	-3.573580	-0.051820	-3.544529
29	H	-4.112095	0.264123	-2.144350

Complex BgPW2 E_{bond} = -5697.36 kcal mol⁻¹ NIMAG: 0

1	C	-3.535460	-0.065528	1.248353
2	C	-3.563749	-0.020054	-1.232730
3	C	-2.844060	-0.093831	2.425661
4	C	-2.855805	-0.043760	-0.014625
5	C	-2.878681	-0.000601	-2.440785
6	C	-1.411611	-0.102543	2.441390
7	C	-1.422355	-0.047529	-0.035074
8	C	-1.480168	-0.003251	-2.465365
9	C	-0.705173	-0.076366	1.203986
10	C	-0.724295	-0.026024	-1.283346
11	C	0.724210	-0.080189	1.203901
12	C	0.743325	-0.030109	-1.283428
13	C	1.430641	-0.110098	2.441230
14	C	1.441399	-0.055320	-0.035235
15	C	1.499176	-0.011756	-2.465536
16	C	2.863121	-0.109071	2.425342
17	C	2.874847	-0.059377	-0.014946
18	C	2.897684	-0.016796	-2.441113
19	C	3.554526	-0.084658	1.247954
20	C	3.582772	-0.039762	-1.233132
21	H	-4.622641	-0.060965	1.247905
22	H	-4.650904	-0.017969	-1.213779
23	H	-3.372533	-0.112636	3.375641
24	H	-3.429641	0.016997	-3.377073
25	C	-0.678684	-0.137885	3.656644
26	H	-0.981099	0.012751	-3.427609
27	C	0.697658	-0.141528	3.656573
28	H	1.000098	0.006747	-3.427731
29	H	3.391592	-0.130613	3.375262
30	H	3.448616	-0.002425	-3.377467
31	H	4.641716	-0.085955	1.247387
32	H	4.669920	-0.043617	-1.214294
33	H	-1.225921	-0.156937	4.595665
34	H	1.244889	-0.163497	4.595535
35	O	0.019891	3.311415	2.359188
36	H	-0.744831	2.737177	2.175367
37	H	0.779694	2.728764	2.181448
